# A Systematic Literature Review on Service Composition for People with Disabilities: Taxonomies, Solutions, and Open Research Challenges

**DOI:** 10.1155/2023/5934548

**Published:** 2023-03-08

**Authors:** Abdallah Namoun, Ali Tufail, Waqas Nawas, Oussama BenRhouma, Abdullah Alshanqiti

**Affiliations:** ^1^Faculty of Computer and Information Systems, Islamic University of Madinah, Madinah 42351, Saudi Arabia; ^2^School of Digital Science, Faculty of Science, Universiti Brunei Darussalam, BE1410, Bandar Seri Begawan, Brunei Darussalam

## Abstract

Integrating smart heterogeneous objects, IoT devices, data sources, and software services to produce new business processes and functionalities continues to attract considerable attention from the research community due to its unraveled advantages, including reusability, adaptation, distribution, and pervasiveness. However, the exploitation of service-oriented computing technologies (e.g., SOC, SOA, and microservice architectures) by people with special needs is underexplored and often overlooked. Furthermore, the existing challenges in this area are yet to be identified clearly. This research study presents a rigorous literature survey of the recent advances in service-oriented composition approaches and solutions for disabled people, their domains of application, and the major challenges, covering studies published between January 2010 and October 2022. To this end, we applied the systematic literature review (SLR) methodology to retrieve and collate only the articles presenting and discussing service composition solutions tailored to produce digitally accessible services for consumption by people who suffer from an impairment or loss of some physical or mental functions. We searched six renowned bibliographic databases, particularly IEEE Xplore, Web of Science, Springer Link, ACM Library, ScienceDirect, and Google Scholar, to synthesize a final pool of 38 related articles. Our survey contributes a comprehensive taxonomy of service composition solutions, techniques, and practices that are utilized to create assistive technologies and services. The seven-facet taxonomy helps researchers and practitioners to quickly understand and analyze the fundamental conceptualizations and characteristics of accessible service composition for people with disabilities. Key findings showed that services are fused to assist disabled persons to carry out their daily activities, mainly in smart homes and ambient intelligent environments. Despite the emergence of immersive technologies (e.g., wearable computing), user-service interactions are enabled primarily through tactile and speech modalities. Service descriptions mainly incorporate functional features (e.g., performance, latency, and cost) of service quality, largely ignoring accessibility features. Moreover, the outstanding research problems revolve around (1) the unavailability of assistive services datasets, (2) the underspecification of accessibility aspects of disabilities, (3) the weak adoption of accessible and universal design practices, (4) the abstraction of service composition approaches, and (5) the rare experimental testing of composition approaches with disabled users. We conclude our survey with a set of guidelines to realize effective assistive service composition in IoT and cloud environments. Researchers and practitioners are advised to create assistive services that support the social relationships of disabled users and model their accessibility needs as part of the quality of service (QoS). Moreover, they should exploit AI/ML models to address the evolving requirements of disabled users in their unique environments. Furthermore, weaknesses of service composition solutions and research challenges are exposed as notable opportunities for future research.

## 1. Introduction

Service composition enables the integration of services into a sophisticated digital service that provides new augmented business processes [1]. This topic continues to fascinate researchers and practitioners, particularly since the number of heterogeneous services and smart objects has risen exponentially. For instance, connected IoT devices are forecasted to reach 50 billion devices worldwide by 2030 [2]. Fortunately, paradigms of service-oriented architecture facilitate the combination of such interoperable components to produce added-value and adaptable services by solving scalability, performance, and security issues, among others [3]. Nonetheless, the empowerment of some particularly deprived user groups remains largely overlooked. This review searches the prospects and challenges of service composition for disabled persons.

Moreover, the number of people classified to have some disability has exceeded one billion globally, equating to a staggering 15% of the world population [4]. Researchers emphasize the importance of making interactive systems and services accessible for users with disabilities. Accessibility is one of the pivotal guidelines of universal design [5]. Moreover, the notion of ambient assisted living (AAL) has emerged to reduce the technical barriers of using modern technologies by senior persons. However, the opportunities offered by pervasive computing and artificial intelligence remain far from being exploited to satisfy the technological needs of people with impairments.

Our survey is motivated by the lack of understanding of the practices, approaches, and solutions that support the seamless composition of services for persons with disabilities. Notably existing service composition surveys, such as[6–8], analyzed composition solutions and tools aimed at creating services for consumption by able-bodied users. Thus, people with disabilities are completely disregarded in these surveys. Important aspects, such as inclusive design practices and accessibility features, are not included in their analysis. Moreover, many of these surveys suffered from methodical shortcomings, such as nonadherence to SLR best practices. On the contrary, our work contributes an in-depth understanding of accessible service composition for people with disabilities, with a focus on the following objectives:

- Develop a taxonomy of accessible service composition encompassing several aspects, such as assistive services, semantic annotations, composition approaches, and execution environments. The taxonomy may be used to assist the designers of software services to understand and tackle the specific requirements of people with disabilities
- Formulate insight into the accessible software services and target users addressed within the scope of service composition
- Provide a comprehensive synthesis of the pivotal service composition approaches for creating integrated services that people with disabilities can access
- Identify and summarize the gaps and open issues pertaining to the area of accessible service composition
- Define future research directions in service composition for disabled people

We organized this systematic literature review into six sections. Section 2 sheds light on the service composition concepts and practices and sets out the motivation for the survey. Section 3 inspects the current surveys and SLRs to clarify the gaps in the literature. Section 4 details the search methodology and highlights the contributions of the survey. Section 5 presents the main findings and discusses the possible implications. Section 6 elaborates on the research problems and acknowledges the limitations and threats to the validity of our survey.

## 2. Background Works

In this section, we remind of the fundamental concepts pertaining to the lifecycle of service composition. In the next section, we present examples of smart and assistive services developed to improve the quality of life of disabled users.

### 2.1. The Key Ingredients

Nowadays, with the rapid growth of technological advancements and the emergence of modern computing paradigms (e.g., pervasive computing [9], IoT [10], and Cloud [11] among others), heterogeneous services have become easily accessible, connectable, and integrable through dedicated web APIs (e.g., [12, 13]), including web services, web applications or mashups, IoT services, mobile services, big data services, and machine learning applications.

Service-oriented architecture (aka SOA) consists of grouping services together, where a service provided by service providers represents the essential part of a service composition process. The features of service composition can be changed by modifying services. A software engineering team typically selects the best collection of services to satisfy the functional and nonfunctional requirements of a system [14]. Service-oriented architecture can be viewed as a software development approach based on replaceable components with standardized interfaces for interaction over standardized protocols [15]. SOA encapsulates the implementation details from the rest of the components, enabling the combination and reuse of components to build complex software packages and ensuring independence from the platforms and development tools [16].

### 2.2. The Foundations of Service Composition

In a service-oriented architecture, the general life cycle of service development consists of a myriad of phases, including service definition, discovery, selection, invocation, composition, deployment, and monitoring [17–19]. Service composition is perhaps the most critical phase in the development cycle where newly added value is produced and offered to potential consumers through the combination of atomic services [20].

**Figure 1:**
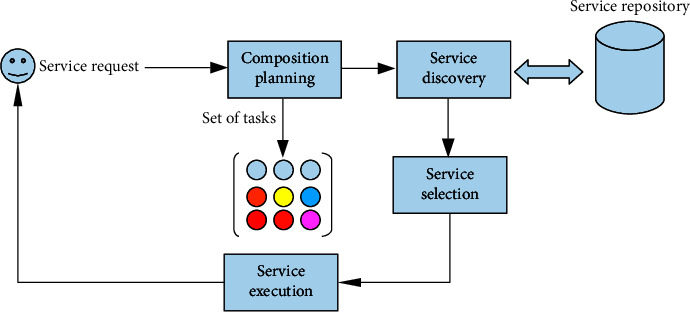
A typical service composition process. This figure shows the main parts and roles of the SOA composition.

Moreover, the service composition process incorporates the subsequent phases, as shown in Figure 1. The first phase is composition planning which aims to specify the requested service and decompose it into a set of tasks. Then, we have the service discovery, which is a search for a service that matches the previously selected tasks. Next, a service is selected among several discovered candidates, and the tasks are executed [21].

The composition process is inherently a challenging process and entails technical complexities. Therefore, significant research efforts have been exerted to understand and facilitate the combination of services by programmers [22] as well as nonprogrammers [23, 24]. Moreover, various tools were developed to integrate data, services, and web resources [25]. To guide service selection for people with impairments, the authors in [26] proposed a machine learning-driven framework taking into account user context and disability factors. However, tools dedicated to empowering the development of accessible services are still rare.

Classically, the composition of services can be either manual or automatic. In manual service composition, an end-user programmer should create an abstract representation of the composition process [27]. In automatic service composition, a composite service specification can be generated automatically by giving a set of component services and a specified requirement requested by the user [28]. Moreover, the assisted composition is proposed as a middle-ground solution between completely manual and automatic approaches [29], where the initial logic is created by a composer and some composition tasks (e.g., selection of compatible services for tasks) are guided by a dedicated intelligence-empowered tool. From another perspective, service composition could be classified into static and dynamic composition [27]. In the static composition, the assembly of individual services is achieved at design time resulting in the creation of static services. On the other hand, in the dynamic composition, individual services are assembled at runtime to deliver a dynamically adaptive composite service based on user profile and context of use.

### 2.3. Ubiquitizing Accessible Services

A related research strand that has gained considerable attention is interactive smart homes [30] and ambient intelligent environments to support the daily activities of the aging population [31]. More relevant to our survey, assistive technologies and smart services that accommodate the needs and constraints of disabled persons have emerged recently to facilitate their quick adoption of ICT [32, 33]. An example of these assistive technologies includes context-aware health services and emergency systems [34]. Another application system employs speech recognition to empower disabled users to operate smart home devices and perform grocery shopping [35]. Typically, such systems incorporate a myriad of IoT devices and sensors (e.g., body position and mobility) that are connected to a federated cloud platform.

Ambient assisted living (AAL) systems refer to the concept of applying computational intelligence (e.g., machine learning) to a designated environment to enable the independent interaction and use of the available technologies and services by older persons [36, 37]. For instance, a smart kitchen was designed to assist the elderly and people with cognitive and physical impairments to use their kitchen appliances (e.g., fridge, oven, washing machine, etc.) autonomously [38]. AAL is unique in its ability to provide assistive IoT technologies, irrespective of their complexity, such as wearable sensors, smart objects, robots, home appliances, mobile devices, and user interfaces for the elderly population [37]. However, it does not necessarily focus on the composition aspects of heterogeneous services and devices.

Assistive technologies and services may be designed to serve two distinct user groups, namely, the older and disabled users. However, these two groups differ with respect to several characteristics and needs. Senior people may experience a decline or loss of physical or cognitive abilities as they age, but it is not always the case. However, disabled persons are likely to have a higher and permanent degree of disability. They usually require constant assistance throughout their life. Since there are a handful of recent surveys on AAL, e.g., [39, 40], the focus of our work is limited to the composition approaches for people with disabilities.

Despite their promising advantages, assistive technologies for disabled persons still encounter various challenges, including the creation of assistive technologies that support independent living [41]. Moreover, a recent survey calls upon the research community to (1) pay close attention to the adaptability of services to the new habits and behaviors of disabled people, (2) be aware of the current context of use and users, and (3) emphasize the need to apply the user-centered design methodology during the design of such services [37]. In the next section, we critically review the existing surveys and pinpoint the research gaps that we aim to tackle in this study.

## 3. Critique of Existing Service Composition Surveys

### 3.1. Motivations for our SLR

Our first logical step was to justify the necessity for a new survey on service composition approaches for disabled users. Our initiative is motivated from two perspectives. First, several dispersed research efforts strived to develop SOA solutions that support the integration of services for disabled users. These works stem from the belief that such special user groups require unique user requirements and considerations than nondisabled users. Nondisabled people are defined as those who do not have any sort of disability or can perform their day-to-day tasks without needing any level of help, including technical assistance and/or human assistance. However, we could not find any survey that integrates these works into one place to help understand the SOA landscape. Therefore, we took the quest to synthesize service composition studies that target disabled people and shed light on the major research challenges in the area. Second, previous findings (e.g., [5, 42]) advocate developing accessible interactive services and systems to empower and facilitate universal design and access. Accessible design refers to the inclusion of the needs of disabled persons (e.g., blind, deaf, etc.) into the software design process [43]. The principles of accessible design also apply to SOA platforms and services. In other words, composite software services and mashups ought to cater to the abilities and constraints of people with various physical and cognitive limitations. Hence, our work is the first effort to pave the way toward assistive service composition for disabled people. We seek to formulate an in-depth understanding of the approaches, algorithms, platforms, and languages of service composition tailored to offer digital accessibility features to assist deprived users in consuming integrated interactive services (e.g., IoT services, web services, and smart services).

### 3.2. Summary of Previous Surveys

We thoroughly investigated 13 recent surveys spanning the area of service composition with a particular focus on end-users who are regarded as “people with disabilities” irrespective of their type or degree of disability. Our goal was to identify research works summarizing existing service-oriented architecture approaches and solutions to create accessible digital services for users with various disabilities. We systematically searched acknowledged databases (e.g., ScienceDirect, Scopus, IEEE Xplore, and Google Scholar) to find relevant review studies and systematic literature surveys. We restricted our search to reviews and SLRs published on or after 2010 using different combinations of search keywords (e.g., “service composition,” “survey,” and “review”) depending on the accepted syntax in each academic search engine. Next, we studied the retrieved surveys and systematic literature reviews to identify the missing research gaps and differentiate our work from the past findings. The idea here was to develop a new understanding that extends the previous knowledge in the research area of SOA. Less relevant SOA surveys, such as [44–46], were excluded from the analysis.  Table 1 compares the prominent surveys in the service composition territory on several metrics, including the focus of the survey, years covered, and major limitations.

Three main remarks can be observed in Table 1. First, none of the existing reviews and SLRs surveyed service composition solutions, approaches, and platforms for the disabled people. Second, most works shifted the focus and application of composition approaches from traditional web environments to cloud, Internet of Things, and ambient intelligence. This is a natural move, given the widespread modern IT technologies. Third, each survey reviewed approximately 20 to 42 articles published between 2003 and 2017. Some surveys missed essential details about their methodology, including the databases used and the number of articles.

As observed in Table 1, prominent SOA surveys were published between 2013 and 2022. These surveys reviewed service composition studies that were published from 2003 to 2017. Many of those surveys covered a publication period ranging from four to six years. In our case, we focused on the articles published between 2010 and 2022, a publication window that is greater than most other surveys in the service composition area. We intended to explore the recent computing approaches that were applied to create accessible SOA solutions. Moreover, our search methodology returned 38 relevant articles, which we believe are enough articles to answer our research questions and draw solid conclusions about accessible service composition. In fact, keeping our SLR focused on this reasonable number of articles enabled us to conduct an in-depth analysis of the selected studies.

The topic of composing assistive services for disabled persons has received little attention, and research studies in this area are scarce. Therefore, to the best of our knowledge, this research is the first survey to explore and synthesize service composition solutions for people with special needs, irrespective of their application domain. Other related areas, such as AAL, are not the main scope of this article since there are several recent AAL-specific surveys already published, e.g., [39]. Moreover, our target population based on the PICO methodology (Table 2) is disabled people, irrespective of age.

### 3.3. Gaps in Literature Findings

Based on the comparison of the prominent surveys in service composition (see Table 1), we infer three significant research gaps that helped us frame our research questions posited in the next section. In our view, previous works failed to address the following aspects:

- **Research gap one:** the lack of in-depth comprehension of service composition approaches, languages, and platforms dedicated to assisting people with disabilities.
- **Research gap two:** the need for service composition surveys that consider the specific needs of disabled users to facilitate the creation of accessible and inclusive composite services. To our knowledge, this work is the first scientific effort to analyze and synthesize empirical studies on SOA solutions for disabled persons.
- **Research gap three:** a substantial weakness in understanding the issues that hinder the integration of accessible services.

To summarize, our systematic survey endeavors to identify and synthesize evidence-based research works that explored composition approaches to simplify the creation of integrated services for consumption by various disability groups. These groups include users disadvantaged by visual and hearing impairments, cognitive impairments, and physical disabilities.

## 4. Systematic Literature Review Methodology

Typically, there are two approaches to reporting review findings, namely, systematic mapping studies and systematic literature review [56]. Although the differences between these two types are subtle, they can be linked to and justified by the genre of the methodology applied by the researchers. In systematic mapping studies (SMS), the researchers generally focus on structuring the research area broadly without evaluating the studies in detail, while systematic literature reviews (SLRs) identify, collect, and synthesize evidence about the main results to answer specific research questions [57]. Hence, we could classify our research work between the two types because we provide a complete synthesis of the related works, yet we do not describe each study separately. We deliberately summarize the key findings and highlight the distinguished studies in our topic of investigation.

**Figure 2:**
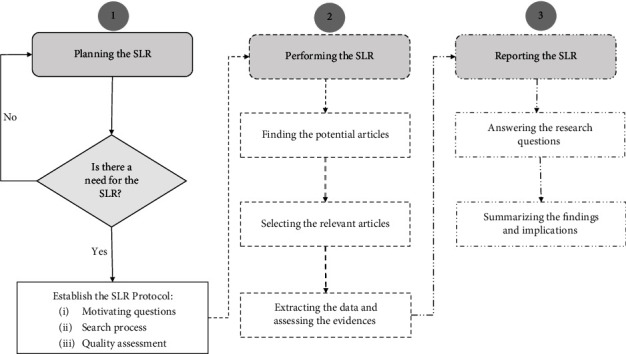
Kitchenham's general phases of the systematic literature review (SLR) methodology. 1 planning phase, 2 execution phase, and 3 reporting phase.

Designing a precise review protocol is pivotal to the correctness and repeatability of a systematic literature review. Therefore, we closely followed and applied the reputable Kitchenham's guidelines in this SLR [58].  Figure 2 depicts three main phases of Kitchenham's methodology. As can be observed, the first phase starts by arguing the need for this survey, followed by positing the specific research questions and establishing the search strategy. The second phase executes the search protocol and applies inclusion and exclusion criteria to determine the most relevant articles to the SLR. The third phase documents the answers to the research questions and highlights the evidence from the selected articles.

### 4.1. Research Questions

The PICO search strategy was adopted to help construct the research questions for our SLR [59]. The PICO model is well known for producing precise and extensive search results. PICO specifies four components, namely, population, intervention, comparison, and outcomes, as listed in Table 2. In summary, our SLR investigates service composition approaches (i.e., intervention) that support the creation of accessible and universal services (i.e., outcome) for users with disabilities (i.e., population). The PICO elements helped us in formulating the search strings. We created equivalent search phrases for each academic database to guide the automated search during the search process.

Moreover, we used the PICO model to produce four motivating questions for our SLR as follows:

1. *Research Question 1 ( ****RQ1****).* What are the characteristics of assistive services mashed up to support the regular activities of disabled persons?
2. This question sheds light on the types and features of assistive services and technologies created to help people with disabilities perform their daily tasks. Further details about these digital services, such as the context of use, user interaction mechanisms, and user devices, would enhance our understanding of the nature of current assistive technologies. Moreover, the question identifies the technical details of composite services concerning the implementation languages, semantics, and ontologies used to incorporate accessibility aspects of disabled users into the composition models.
3. *Research Question 2 ( ****RQ2****).* To what extent are disabled users involved in designing composite services?
4. This question determines the genre of disabilities addressed and the extent to which disabled people were consulted during the assistive services composition process. Their involvement could be linked to the design and/or testing of aggregated services. Moreover, this question investigates the accessibility and social needs of people with diverse disabilities when developing assistive services.
5. *Research Question 3 ( ****RQ3****).* What are the service composition approaches and algorithms developed to incorporate the needs of people with disabilities and accessible design guidelines while creating new assistive services?
6. This question collates the approaches through which assistive services are aggregated, focusing on composition notations, components, paradigms, algorithms, and so on. In our work, this technical knowledge is encapsulated within a novel seven-facet taxonomy to aid assistive service development researchers and practitioners.
7. *Research Question 4 ( ****RQ4****).* What are the open challenges hindering the integration of accessible design within service-oriented architecture solutions?
8. This question summarizes the major problems that deter the development of accessible services for disabled users. These problems will serve as a practical road map for other researchers to focus their future works in a bid to satisfy the digital needs of people with disabilities.

### 4.2. Search Strategy and Process

The search process was initiated by scoping the search terms, which were derived from the PICO elements and aligned with the abovementioned research questions. As shown in Table 2, synonyms of our target population and intervention were used to expand our results, as recommended in [60]. Boolean operators (AND and OR) were used to link the search keywords and facilitate the search in the selected databases.

The composite search terms were constructed and used to find potential articles in six academic databases, including ACM Digital library, IEEE Xplore, ISI Web of Science, ScienceDirect, Springer Link, and Google Scholar. Other bibliographic resources, such as DBLP and CiteSeer, were not searched since their results are already included within six major databases. Our protocol-driven searches looked up the title, abstract, and keywords of articles in all databases except for Google Scholar, where only the title was searched. This was because the full search of text in Google Scholar would return a huge number of articles. Moreover, our search was restricted to service composition studies published between 2010 and 2022. This was when pervasive computing and IoT services gained momentum and popularity.

We kept our search focused on the last 12 years and applied a comprehensive and meaningful filtering mechanism considering the various SLR guidelines, resulting in a reasonable and manageable number of articles (38 selected articles). This enabled us to carry out a thorough analysis of each article. Moreover, several studies have mentioned ten years window as an appropriate period to retrieve the most relevant research articles in the field of interest. For example, the study published by [61] uses a ten-year time frame, i.e., 1995–2005, as the most appropriate window to search for relevant articles. Similarly, a more recent study [62] also uses ten years as the most appropriate time frame to search for relevant studies.

Admittedly, the territory of service-oriented architecture for people with disabilities is not clearly defined nor well understood. Therefore, the authors resorted to other search techniques, mainly manual search, to strive for full coverage of the research area. We also applied (1) backward and (2) forward snowballing, which refers to using the list of references or citations of the primary studies to identify additional candidate studies [63]. The guidelines and processes elaborated in [61] were shadowed to optimize the search results. Although such manual approaches may be thought less efficient, the literature backs up their usefulness in pinpointing relevant articles [61]. In our case, the newly discovered articles were added to the list of articles.

**Figure 3:**
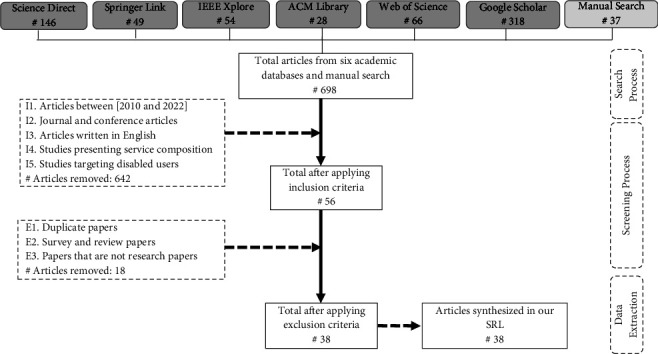
Search and selection results of our SLR. This figure shows the number of articles retrieved from each phase and enumerates the inclusion criteria (denoted: I) and exclusion criteria (denoted: E) used to choose the final studies.

Overall, the search exercise collated 698 candidate studies, where 661 articles emerged from the protocol-based search (i.e., academic databases) and 37 supplemental articles from manual searches.  Figure 3 succinctly sums up the search and selection results. It is worth noting that the supplementary articles were derived from scanning the relevant articles (i.e., snowballing) and hand searching to reduce the bias during the selection process. In Figure 3, we bundled the manual search results with the total candidate articles to facilitate reading the figure.

Due to the versatility of search options provided and the limitations imposed by each academic database, we had to use a fuse of techniques, where sometimes we had to (1) split the queries into small search phrases and (2) search the title, abstract, author-specific keywords, or complete article to identify a reasonable number of articles. We provide more insights into the queries that we used to identify the most relevant articles from each database.

For ScienceDirect, service composition aspects were searched in the title, abstract, or author-specified keywords, while the disability aspects were searched in all parts of the articles (metadata and full text). In IEEE Xplore, we deployed our queries using AND/OR operators and searched the metadata and full text of articles to obtain our desired results. For the Springer database, we searched for service composition aspects in the titles of the articles, while the disability aspects were searched in all parts of the articles. For the ACM digital library, we searched for service composition aspects in the abstract of the articles only and for disability aspects, we searched anywhere in the articles. For the ISI Web of Science, we used the Query builder option in the advanced search and submitted queries for service composition aspects and disability aspects to search all parts of the articles. Finally, for Google Scholar, service composition-related concepts were searched within the titles of the articles, while disability aspects were searched in the full text of the articles.

### 4.3. Selection Criteria

When we implemented the search strategy, we retrieved 698 possible articles. Our subsequent task was to refine the results into a subset of studies that assisted in answering our research questions. Therefore, explicit inclusion and exclusion criteria were defined and applied to shortlist the relevant articles. We specified and applied five inclusion rules and three exclusion rules, which are represented via the dotted rectangles in Figure 3. In summary, only peer-reviewed, English-written journal and conference articles published between 2010 and 2022 were filtered from the six bibliographic databases.

Next, the authors carefully read the title and abstract of each article to decide on the relevance of the candidate studies. They had to read the full text in certain instances, particularly when the inclusion decision was not possible from reading the title and abstract alone. For any study to qualify for inclusion in our review synthesis, it must satisfy two conditions. First, the study ought to investigate service composition or integration models, techniques, or approaches. Second, the study must target the creation of software services for people with disabilities regardless of the form of disability.

Research articles that were duplicates and appeared in multiple academic databases were disregarded. In the case of multiple publications from the same authors about the same study, only the latest and most comprehensive article was included in the analysis. Review and survey articles were omitted from our SLR. Furthermore, articles that focus primarily on other aspects of the software service lifecycle, such as discovery, selection, invocation, and monitoring, were excluded.

Upon applying the inclusion and exclusion criteria (in Figure 3), the pool of articles was reduced to a reasonable number of 38 relevant articles, constituting the final list of our SLR. Indeed, this number of articles is in the range of existing service composition surveys (e.g., [47, 48]).

### 4.4. Quality Assessment

We carefully devised the assessment criteria to evaluate the quality of the primary studies that qualified for our SLR. Quality assessment is conceived as a crucial appraisal strategy of the collected evidence since it helps to confirm the strength, thoroughness, and credibility of the selected studies [64, 65]. In total, we devised 17 criteria covering distinct research aspects (e.g., research questions, methodology, data, user testing, and findings) to realize an exhaustive quality assessment of the selected studies. The criteria listed in Table 3 were evaluated on a three-point scale using the following scores (Yes = 1, No = 0, and Partial = 0.5). The quality score for each study was calculated by totaling the individual scores awarded to each of the 17 criteria.

### 4.5. Data Extraction and Synthesis

**Figure 4:**
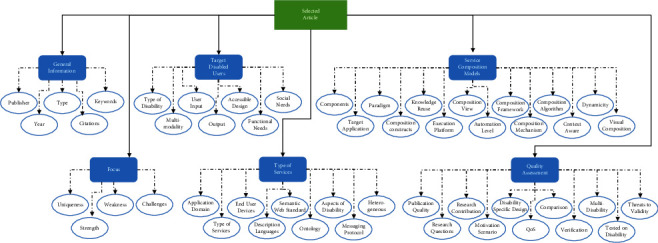
A high-level overview of the data extraction form, where form subsections are linked to our research questions.

In our analysis of the articles, we developed a comprehensive analysis form following the frameworks proposed by [6, 22, 49], covering major aspects of service composition. All results are discussed thoroughly and summarized in the proposed taxonomy in the results section. The form was constructed to guide and aid the extraction process of the required data to answer the SLR questions.  Figure 4 lays out a high-level structure of the data extraction form, which incorporates six main sections. These sections encapsulated general information about the articles, the focus of the studies, target disabled users, service composition models, type of services, and quality assessment. Each of these subsections incorporated further details about SOA. Remarkably, the service composition models subsection was constructed based on the recommendations offered in recent works, precisely in [6, 22, 49]. The authors in [22] advocate using their analysis taxonomy, which was constructed to acquire an in-depth understanding of service composition models, techniques, and tools.

Although the process was exhaustive, the extracted data assisted us in answering our research questions. It is worth noting that the form was refined several times through pilot data extraction before reaching the final state presented in Figure 4.

## 5. Results and Discussion

This section succinctly synthesizes the main findings and evidenced observations from the primary studies of service integration for disabled persons. The results are presented in five subsections as follows.

### 5.1. Service Composition Landscape

**Figure 5:**
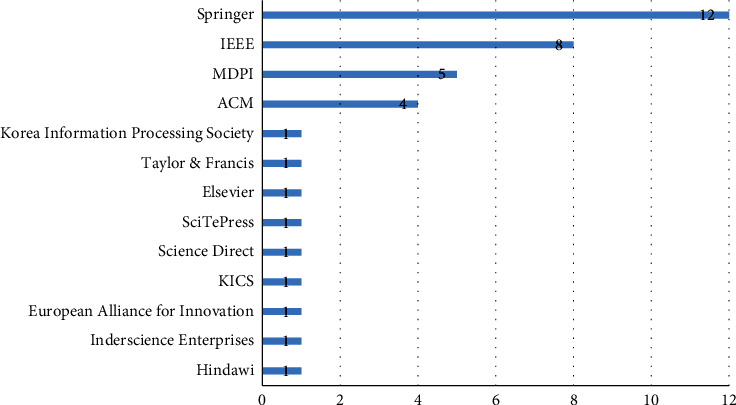
Distribution of articles per publisher. IEEE = Institute of Electrical and Electronics Engineers, ACM = Association for Computing Machinery, MDPI = Multidisciplinary Digital Publishing Institute, and KICS = Korean Institute of Communications and Information Sciences.

We began by inspecting some general information (i.e., publication timeline, publishers etc.) about the articles selected in our SLR.  Figure 5 shows that most studies (76%) were published in four popular venues, namely Springer, IEEE, ACM Digital Library, and MDPI. The remaining studies (24%) were scattered across other academic publishers (e.g., Taylor and Francis, ScienceDirect, and Hindawi). Out of 38 peer-reviewed articles, 20 were presented at conferences (52%), and 18 were published in journals (48%). All articles appeared in distinctive venues (i.e., no two articles were published in the same venue).

**Figure 6:**
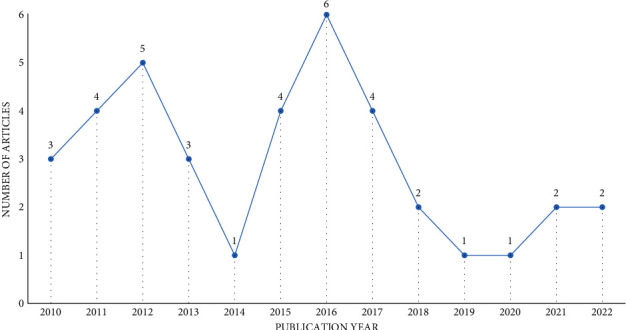
Number of articles published from 2010 to 2022.

Figure 6 shows the trend of research articles published over the past 12 years (from 2010 to 2022). The graph shows an apparent decline in the number of published research efforts concerning SOA solutions tailored toward assisting disabled persons in the past four years. This phenomenon is intriguing, particularly with the prevalence of modern enabling technologies such as IoT, machine learning, robotics, and augmented reality technologies.

**Figure 7:**
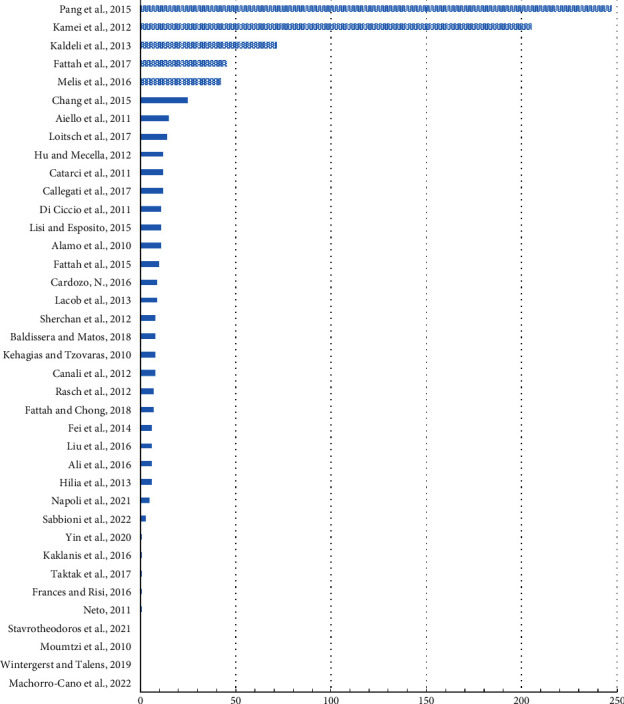
Number of citations per article from Google Scholar. Studies with more than 40 citations are represented using patterned bars.

Figure 7 shows that 15 articles (39.47%) were cited at least 10 times. The highly cited studies (i.e., >40 citations) that were arranged in descending order include [66] in Enterprise Information Systems, [67] in IEEE Network, [68] in ACM Transactions on the Web, [69] in Sensors, and [70] in IEEE International Smart Cities Conference. These top-cited studies were published between 2012 and 2017, where [66, 67] were arguably the most impactful works in service composition for accessible services. The authors in [66] explore the domain of in-home healthcare services that are based on Internet of Things technology. With the help of a codesign framework, the authors attempt to integrate devices, services, and information systems to improve the quality of life of the elderly and disabled users. However, the authors in [67] emphasize the notion of cloud networked robotics where the integration of standalone robots and their functionalities is accomplished to provide seamless support for the daily activities of people with varying disabilities (e.g., elderly and disabled). Six services were considered in the baseline project; however, the presented study focused on touring services for a physically disabled person in a shopping mall.

**Figure 8:**
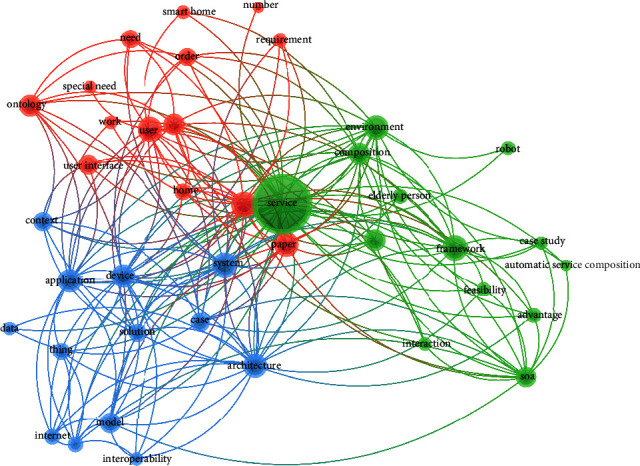
The co-occurrence network map of articles' terms (appearing in titles and abstracts). The figure shows three distinguishable clusters of key terms.

We carried out a co-occurrence analysis of articles' keywords to understand the common concepts that were researched in our selected articles. The first analysis reports the frequency of the authors' keywords used in the articles. A total of 171 keywords were collated from the 38 articles (on average, 4.5 words per article). The most recurring keywords in the articles included service composition (16 times), service (13 times), Internet of Things (8 times), objects (8 times), SOA (6 times), user interface (6 times), web (5 times), ambient assisted living (3 times), and context-aware (3 times). Moreover, we created a term co-occurrence map for the terms appearing in the titles and abstracts of our final articles.  Figure 8 depicts all the concepts, their frequency, and the co-occurrence between these concepts (in the form of links). Three cohesive clusters were evident from the visual representation, namely, (1) services and service composition, (2) users and their requirements, and (3) IoT solutions and devices.

### 5.2. Target User Groups

**Figure 9:**
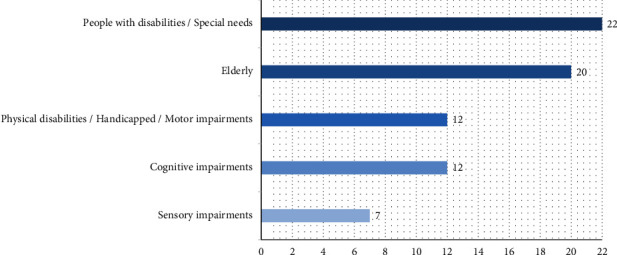
Type of user groups and disabilities addressed and number of studies (*X*-axis = the number of studies).

This subsection describes the characteristics of users with diverse disabilities who were targeted in the selected studies through the design and/or validation of appropriate SOA solutions. Moreover, it unveils the genre of software services and user interaction mechanisms used to enable the consumption of composite services. Our results show that it was not uncommon for one research article to compose services that assist multiple user groups. 26 articles targeted more than one user group; for instance, the authors in [71] addressed three user groups: the elderly, users with visual impairments, and users with mental impairments. Each study targeted between one and four types of disability. It was quite noticeable that elderly users were also considered when creating assistive composite services. The older generation represents a viable target group for assistive composite services since older adults suffer a drastic reduction in their cognitive and physical abilities as they age.  Figure 9 depicts a taxonomy of the highly recurrent user categories that were targeted in the SLR studies, including (1) people with disabilities (appeared in 22 studies), (2) elderly persons (20 studies), (3) people with cognitive impairments (12 studies), (4) people with motor impairments (11 studies), and (5) people with sensory impairments (7 studies).

**Figure 10:**
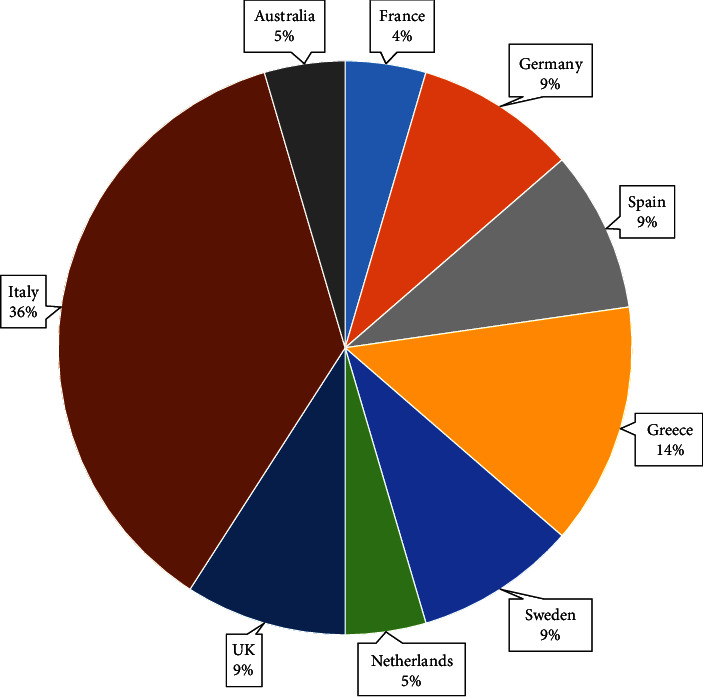
Countries of people with disabilities involved in the studies. The figure shows that European countries (with Italy in the lead) represent the majority.

The “People with disabilities” category represents a general group where the authors did not specify the type of disability targeted in the study, i.e., the solution was aimed at people with special needs. Cognitive impairment represents a loss of functions related to the brain processes (e.g., memory, attention, and understanding). In contrast, sensory impairments represent disabilities linked to the vision (i.e., blind) or/and hearing (i.e., deaf). The ethnicity of target user groups was mainly European (36%) and Australian (3%), while the remaining (61%) were unspecified. Inspecting the countries reported in the articles, the end-users who were involved in the service integration studies were from Italy [68, 72–77], Greece [77–79], UK [77, 79], and Sweden [66, 79], as depicted in Figure 10. It is worthwhile to note that three studies involved users from multiple countries (i.e., [77–79]).

Based on age, three classes of user groups emerged from the syntheses, specifically the elderly and adults (11 studies), elderly (7 studies), and elderly and children (2 studies, specifically [80, 81]). However, 17 articles (44.73%) remained anonymous about the age group of their end-users.

The authors in [82] emphasize the need to advocate for disabled persons in the design process to cocreate socially inclusive systems. However, we observed that only eight studies (22.22%) opted to consult with end-users regarding their universal design decisions. The number of users engaged in the studies varied considerably between 1 and 1958, as depicted in Table 4. Notably, the study [79] engaged a high number of end-users (1958 users) in the evaluation process of their service composition approach. The proposed method combines ambient assisted living services and prepares them for consumption by older people with cognitive impairment, especially in emergencies. Only two articles [68, 85] gave gender-specific information.

When we inspected the modalities used for interaction between the users and composite services, 19 studies (52.77%) reported the implementation of multimodality to facilitate the use of services. In comparison, three studies relied on unimodality (a single interaction modality). Conceptually, multimodality refers to using different modes (e.g., aural, visual, and haptic) to enable interaction with assistive services [86]. 14 articles were unclear about how interaction can be conducted. The next natural question that we attempted to discover concerns the techniques and devices used during the interaction process.

**Figure 11:**
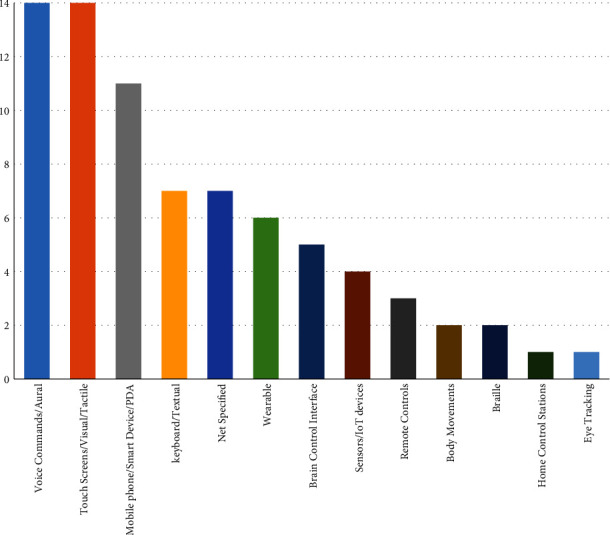
Frequency of input techniques and devices. Voice and tactile interaction prevailed as the major types of user interaction.

Three input techniques and devices emerged as popular choices among the selected studies (see Figure 11). Both speech recognition commands and tactile interaction appeared in 14 studies (36.84%). Moreover, (smart) mobile phones or devices were reported in 11 studies. In six studies and five studies, wearable technologies and brain control interfaces were mentioned. Only a few studies used body movements (i.e., [87, 88]) and eye-tracking technology (i.e., [74]) to enable user interaction.

**Figure 12:**
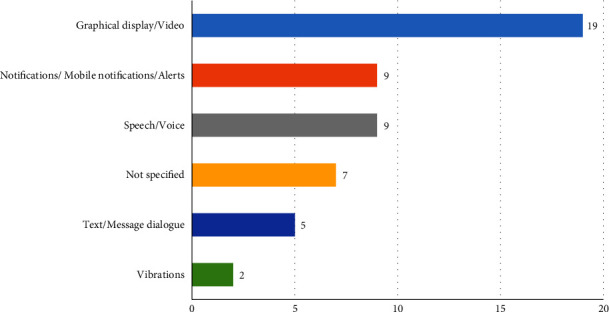
Frequency of output techniques and devices (*X*-axis = the number of occurrences).

Screen displays were the primary type of output device for delivering information and feedback to people with disabilities (19 studies). This was followed by speech (9 studies) and notifications/alerts (9 studies). Text messages and vibrations were used less frequently, as shown in Figure 12. One of the critical aspects that we investigated relates to the accessibility of SOA solutions and the services proposed in the selected studies. Strikingly, only 18 articles (47.36%) considered accessibility features of the composite services to enable the inclusion of people with disabilities. Nine studies did not consider the accessibility of their services, while another nine articles remained unspecific about designing for accessibility.

Based on the recommendations of [82], accessibility design must be inspected from two perspectives, i.e., functional and social aspects, to create socially accessible designs. The functional factors investigate the technology features such as information architecture that improve the technical quality of a system. In contrast, the social design factors impact user perception and use of the system, such as personal safety, respect, appropriateness, emotional support, and social appeal.

Twenty studies highlighted functional features that should be considered in developing assistive technologies. In general, these studies suggested the use of multiple input and output modes of interaction (e.g., [74, 89, 90]), the adaptation of interfaces to fit diverse device characteristics (e.g. [73, 91]) and user profiles (e.g., [81]), and the simplicity of the user interface (e.g., [78, 79, 83]). Most of the functional needs were related to the interaction and user interface of composite services. On the other hand, we were surprised to discover that only five studies (13.15%) incorporated social needs when mashing up services (see Table 5).

### 5.3. Services and Domains of Composition

**Figure 13:**
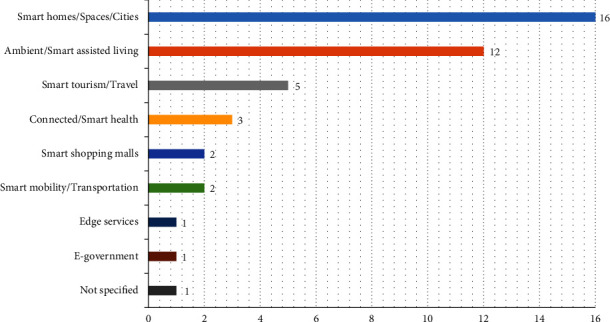
Application domains of composite services (*X*-axis = the number of studies). Most composition solutions targeted (1) smart homes and cities and (2) ambient assisted living.

Next, we inspected the targeted application areas in the selected studies. Two major domains emerged from the analysis. 16 studies provided composite services to enable the realization of smart homes (e.g., [76, 93]), spaces (e.g., [88]), and cities (e.g., [70, 73]), while another 12 studies aimed at creating composite services to support the concept of smart assisted living (e.g., [84, 94–96]) as depicted in Figure 13. Other emerging fields of application include smart tourism (e.g., [72]), smart health (e.g., [66, 97]), and smart transportation (e.g., [74]). The integrated services in these domains were accessed and consumed using web systems (18 studies), smart mobile devices (15 studies), and sensory environments (11 studies). Sensory environments refer to places and spaces designed to support the processing of sensory information through multiple senses to enable disadvantaged groups, such as children with autism [98], to partake in several activities and improve their well-being. In two distinguished instances (e.g., [67, 92]), services were invoked via robots.

Overall, two types of services were composed, namely, software and web services (28 studies) and IoT services (16 studies). Most compositions included heterogeneous services (33 studies, 86.84%), while only five articles were unspecific about the composed services (e.g., [83, 88, 89, 95]). Only two articles made their services publicly accessible (e.g., [81, 99]), while the remaining 34 studies completely concealed the details of their services. The composite services were accessed through a myriad of interactive devices ranging from smartphones to devices (27 studies) such as PDAs, desktop computers (7 studies), brain-controlled devices (3 studies), wearable technologies (3 studies), smart wheelchairs, and smart sticks (2 studies). However, seven studies remained vague about which devices are used by disabled persons to benefit from the composite services.

In the next step, we inspected the languages used to describe the functionalities of services. Only 13 studies described services using WSDL (6 articles, [71, 72, 75–77, 87]), REST (4 articles, [68, 69, 96, 99]), and BPEL (3 articles, [89, 100, 101]), while 25 studies did not disclose details about their service description languages. To facilitate the composition activities, services were semantically annotated in 15 studies (39.47%), unannotated in 7 studies (18.42%), and unspecific in 16 studies (42.10%). Semantic technologies, e.g., RDF and OWL [102], were utilized to model and encode meanings and relationships independently from service implementations to enable machine interpretation [103]. Such abstraction of semantic descriptions achieves critical benefits, including interoperability, automation of service lifecycle tasks, and improved performance.

A myriad of ontologies is proposed to remove the barriers to using technologies for disabled persons ([104, 105]). Ontological models and taxonomies may be used to express diverse aspects of a disability, including the type of disability, interaction context, abilities, and policies, among other entities. However, a recent review revealed a lack of applying semantic web technologies to realize wider software accessibility [106].

In our survey, only five studies gave details of the semantic technologies that were employed to annotate the services, specifically BCDL0 [95], Composite Virtual Object [96], Ontology Slice (Graph) [107], SWRL [93], and WSMO [71]. Inspecting the ontology frameworks and languages used in our synthesis showed the dominance of OWL-S (8 studies [69, 72, 77, 79, 93, 96, 99, 108]). OWL-S is a semantic markup ontology built on top of DAML-S, originated to describe the profile, operations, and interoperability aspects of services [109]. There were other ontologies used, such as WSMO and WSML [71], PROV-O [80], and OpenRDV Sesame [96]. Exploring the presented ontologies showed that the authors focused on modeling the type of impairment (12 studies), user details and profiles (12 studies), physical context (10 studies), input (8 studies), and output modalities (5 studies). It was not uncommon for some studies to model multiple aspects of disability, such as [66, 71, 74, 85, 91]. On the other hand, 16 (42.10%) studies did not model any disability aspect.

With regard to messaging protocols, five studies (i.e., [75–77, 83, 87]) used Simple Object Access Protocol (SOAP), two studies (i.e., [93, 96]) used Representational State Transfer (RESTful), and 31 articles remained unclear about the way communication occurs between the composed services. The SOAP and REST communication protocols differ since the SOAP standard exposes the service logic and operations using dedicated XML-based interfaces. In contrast, RESTful services use HTTP to access web resources and facilitate communication among services. There are various reasons why world-renowned companies prefer RESTful architecture, primarily owing to the ease of integration with other resources, speed, and decreased bandwidth. Moreover, only one study [77] provided Universal Description, Discovery, and Integration (UDDI) support to enable the registry and discovery of accessible services by clients and composers.

### 5.4. Service Composition Approaches

The composition brings various types of software components together to serve business requirements. In our SLR, the services integrated ranged between data (20 studies), application logic (16 studies), and user interface components (1 study [81]). The services communicated mainly by using SOAP (5 studies), REST (4 studies), and Open Service Gateway Initiative (OSGi) (1 study, [100]). In six articles [71, 72, 75–77, 87], services were described using Web Services Description Language (WSDL). Other less frequent description languages that were reported one time only included HTTP Rest [96], BPEL [89], and deployment descriptors [100]. However, 27 articles did not reveal details about the languages of their services. Data between services are exchanged in the form of XML/JSON (14 studies, 36.84%) and Java Objects (1 study).

**Figure 14:**
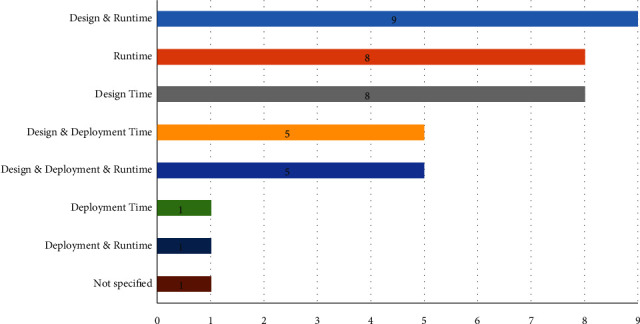
Service selection time during service composition (*X*-axis = the number of studies).

Most services implemented the pull technology and/or business protocol (22 studies) as their primary interaction style. In the pull process, service clients periodically submit requests for the services. On the other hand, the push process sends updates to the clients as information becomes available. However, business protocols outline the rules for sending or receiving messages between services. Service selection for compositions may take place during design time, deployment time, or runtime. In our pool of studies, it was more frequent for services to be selected during design time and/or runtime (25 studies, 65.78%) than deployment time (e.g., [72]), as shown in Figure 14. Only [95] did not indicate the time-of-service selection.

**Figure 15:**
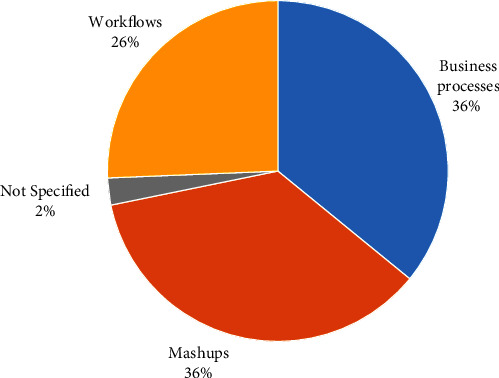
Target application of compositions. Mashups topped the list of composite applications.

The studies targeted creating three types of composite applications, i.e., mashups, business processes, and workflows. In essence, mashups fuse a mix of web content, resources, and applications from multiple sources into a single web application. Business processes refer to the logic and steps that are responsible for executing business rules and activities of a service. Workflows, however, refer to a repeatable and sequential set of tasks, defined as part of a formal diagram, to achieve a particular process. In our SLR, mashups (14 studies) were the most produced type of applications, followed by business processes (14 studies), and workflows (9 studies), as depicted in Figure 15.

**Figure 16:**
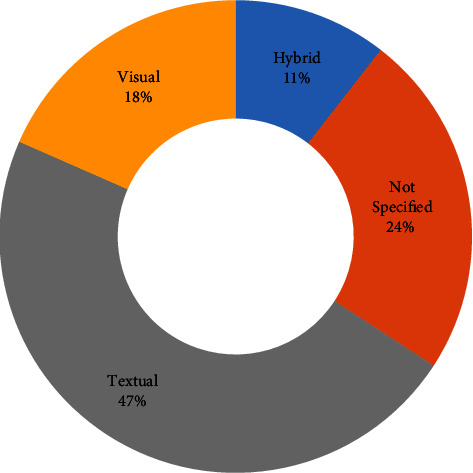
Distribution of composition notation types in our SLR.

Text approaches (18 studies, 47%) were used to represent compositions, followed by visual notations (7 studies, 18%), as shown in Figure 16. The text notations were highly reliant on XML and code-based approaches. In contrast, the visual notations combined diagrams and spreadsheets to represent services and their underlying logic. Four articles (i.e., [73, 75, 76, 110]) reported the use of hybrid (text and visual) notations. Nine articles remained anonymous about the composition notation they employed.

**Figure 17:**
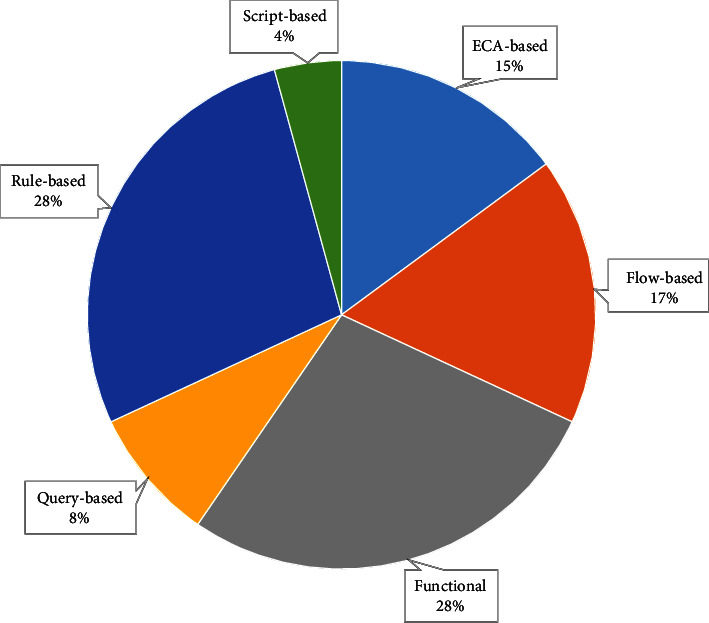
Composition paradigms emerging in our SLR (ECA = event condition action).

Next, we inspected the composition paradigms that the researchers adopted. According to [22], a composition paradigm is a programming approach that uses dedicated principles to solve problems. Our synthesis demonstrated the prevalence of functional (13 occurrences) and rule-based (13 occurrences) approaches (as shown in Figure 17). In functional paradigms, services are represented in the form of stateless functions. In rule-based paradigms, services are represented as conditions and business rules that map conditions into actions. This was followed by the flow and event condition action (ECA)-based approaches (7 occurrences each). Flow-based paradigms expose services as black boxes connected within a graph, and they usually include control and data flow approaches. The query and script-based approaches were the least favorite paradigms among the authors. Eight studies (i.e., [67–69, 71, 83, 85, 94, 107]) mixed more than one composition paradigm to integrate services.

We looked at the building blocks of composite services. Reference [22] divides composition constructs into process-oriented patterns, data flow patterns, and data transformations. Control flow constructs (24 studies, 66.66%) were used more frequently than data flow constructs (10 studies, 27.77%). This is probably justifiable since data-flow concepts are usually more difficult to interpret than control-flow concepts [24]. In control flow patterns, the order of executing services and activities is specified, whereas, in data flow patterns, data passing from one service to the next is defined. Composition concerns that were considered included security (8 studies, [66, 75, 76, 85, 91, 92, 96, 100]), quality of services (5 studies, [68, 71, 83, 84, 107]), and exception handling (3 studies, [68, 70, 90]). One of the key advantages of SOA is its ability to promote the reuse of artifacts and techniques.  Table 6 shows that service components (20 studies) were the most reused artifact, while search and discovery were the most reused technique (16 studies). Nine articles remained completely unspecific about knowledge reuse.

Concerning the automation of composition, model-driven composition (16 studies) emerged as the dominant type, followed by synthesis-based (11 studies) and planning-based development (6 studies). Automating service composition refers to the automation of the tasks of composition workflows (e.g., discovery, selection, and binding of services). In model-driven development, software models and formal diagrams, such as UML, state machine, and BPMN, were used to generate code and automate service composition. On the other hand, synthesis-based composition, such as the Roman model, followed a client-based approach by exploiting the behavioral features of services to synthesize new compositions via a community orchestrator [112]. Lastly, planning-based composition focused on rule-based reasoning and reinforcement learning, where the composition was specified as a set of conditions and preferences. Notably, no study provided tool support during the composition of accessible services.

**Figure 18:**
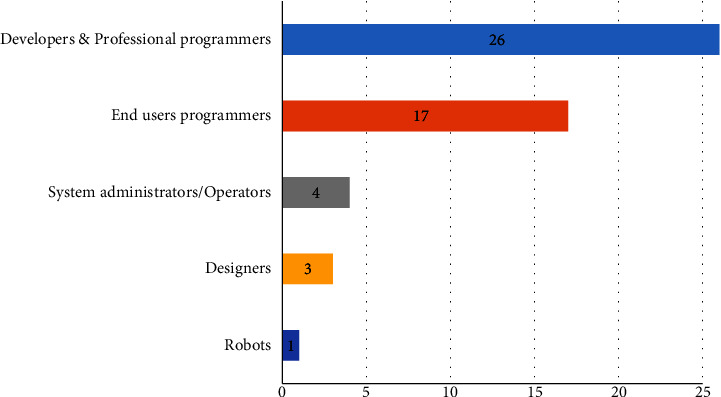
Target end-users of composition tools and environments of accessible composite services (*X*-axis = the number of studies).

Accessible services were deployed and executed mainly on premises (26 studies, 68.42%) as opposed to the cloud (4 studies, [71, 74, 78, 110]) or cloud and premises (3 studies, [66, 77, 92]). The business process engine (i.e., BPE) was the most reoccurring runtime engine for executing processes and services (13 studies), followed by the service bus (4 studies) and code generation (3 studies). Overall, the service composition tools were developed to be operated by two main types of users, namely, professional programmers or developers (26 studies, 68.42%) and end-user programmers (15 studies, 39.47%), as shown in Figure 18. End-user programmers refer to ordinary people who have no professional software development education or expertise [20]. Naturally, they are nontechnical users or domain experts. Interestingly, [88] presented a service composition framework that automates task planning by service robots in smart spaces equipped with information sensors.

**Figure 19:**
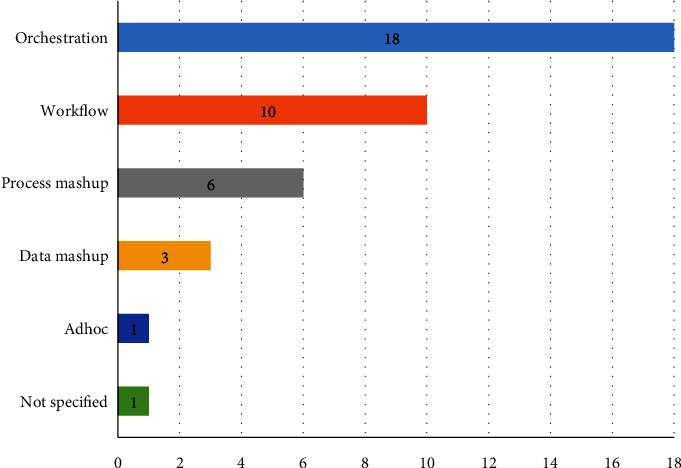
Frequency of composition views in our SLR (*X*-axis = the number of studies). Orchestration and workflow views topped the chart.

Service composition could be achieved in different forms [25]. The composition view refers to the perspective and focus of the composition approach (i.e., processes and data) [113]. In our systematic survey of accessible service composition, service orchestration (47.36%) and workflow (26.31%) compositions were the most frequent composition approaches, as depicted in Figure 19. Service orchestration is usually regarded as a single party. It enables the management, streamlining, and execution of business processes by invoking the correct services for the different processes. However, workflow compositions are based on a progressive flowchart-like diagram of tasks and actions needed to accomplish specific goals. Workflows might also include some technical descriptions while remaining are platform-independent. Mashup composition (23.68%) comes next in the list where users can create a process (e.g., [69, 71, 83, 84, 94, 99]) or data (e.g., [72, 77, 88]) web applications using a dedicated dashboard.

25 studies (65.78%) applied automatic composition of services, 12 studies (31.57%) applied semiautomatic composition, and one study (2.63%) applied manual composition [94]. The selection and binding of available services occurred during runtime (10 studies, 26.31%), design time (7 studies, 18.42%), and in both times (i.e., dynamic and static) (16 studies, 42.10%). Only 25 articles (65.78%) gave details about some form of verification or validation of the quality of their service composition approaches.

**Figure 20:**
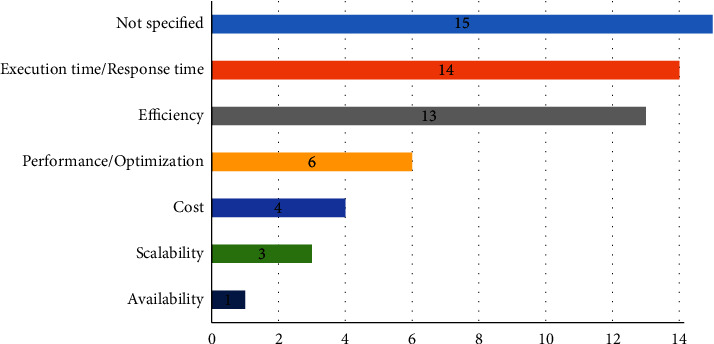
Functional quality dimensions considered during the composition of accessible services (*X*-axis = the number of studies) in descending order.

Quality of service (QoS), or otherwise known as service properties [114], were modeled in 28 studies (73.68%). However, 10 (26.31%) studies (i.e., [67, 72, 75, 76, 85, 95, 99, 101, 110, 115]) did not model the overall performance of services. Out of the 28 articles, only 13 interesting studies delved into the disability characteristics that were considered during the design of their SOA solution. The accessibility quality factors that were reported included the type of disability (i.e., [74, 77, 80, 91, 97]) and user interface features, such as usability, interactions, adaptability, and simplicity (i.e. [68, 71, 78, 79, 83, 89, 92, 94]). However, 20 out of 28 articles considered seven functional properties during service composition.  Figure 20 depicts the most frequent functional QoS, namely, execution and response time (12 studies), efficiency (11 studies), and performance/optimization (6 studies) of composite services. Other service quality dimensions emerging from the studies included cost, scalability, context information, and availability.

Concerning service composition tools and frameworks, SM4All appeared in all 6 studies (i.e., [68, 73, 75, 76, 87, 111]), Business Process Execution Language (BPEL) in two studies (i.e., [89, 100]), and Web of Objects in two studies (i.e., [96, 108]). SM4All stands for smart homes for all, where smart homecare services are immersed and integrated into a dynamic environment using semantic technologies to serve the demands of elderly and impaired users [75]. Among other service composition frameworks were OSGi [100], Cloud4all Service Synthesizer [99], iMedBox [66], MicroApp Generator [81], Puglia@Service [72], SCoPE [85], and SCORPII [110].

Most studies (81%) implemented a framework-based mechanism to compose services. However, there were a few alternatives to this composition mechanism; for example, [79, 107] implemented heuristics to compose services, while [80] used agents to facilitate the composition tasks. Half of the articles (50%) did not provide details of their service composition algorithms and approaches. In the remaining studies, multiple objective approaches [69, 75, 76, 78, 83, 84, 88] and AI/ML techniques (e.g., collaborative filtering [79]), reinforcement learning [107], and graph plan algorithm [93] were used the most (41.44%), except ant colony optimization [74] and the fuzzy preference model [110].

30 (83.33%) studies attempted to capitalize on context awareness while combining services. On the other hand, a few articles [66, 77, 92, 94, 99, 100] did not consider the context during the composition. However, only 20 (55.55%) articles considered the dynamicity and uncertainty aspects of the environment during the composition process. Interestingly, only five studies [72, 77, 81, 88, 93] provided support for visual composition.

### 5.5. Accessible Service Composition Taxonomy

Taxonomies represent a classification structure of knowledge or key concepts in a particular domain [116]. Arguably, taxonomies contribute various advantages, including the organization of knowledge into a logical structure, categorization of concepts/classes, and creation of a common vocabulary of the topic concepts [116]. However, developing effective taxonomies is not an easy task. The literature proposes several ways to represent taxonomies, including a hierarchy, tree, paradigm, or faceted analysis. Our research argues that the proposed taxonomy follows a facet-based structure where different concepts are represented independently through multiple perspectives. This taxonomy type can be easily altered and/or extended in subsequent research studies.

**Figure 21:**
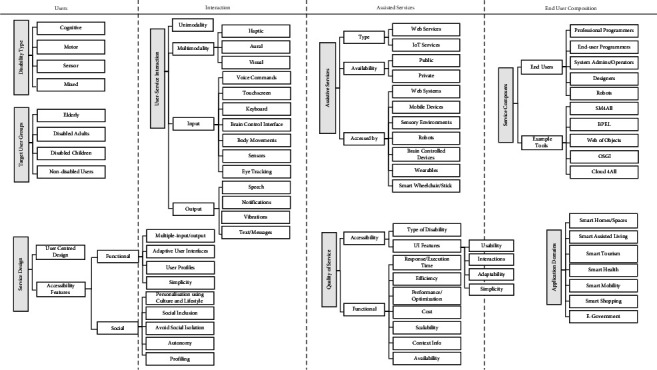
Accessible service composition taxonomy-users, interaction, assisted services, and end-user composition.

**Figure 22:**
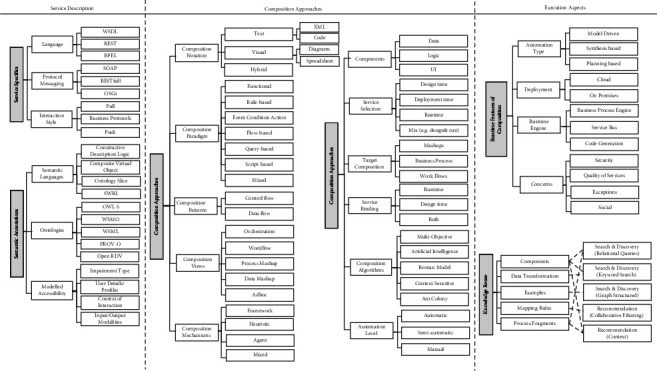
Accessible service composition taxonomy-service description, composition approaches, and execution aspects.

We developed the so-called “accessible service composition” taxonomy in this SLR. This taxonomy concerns the classification of several aspects of service composition for people with disabilities. It aims to assist the developers to create accessible services that fit the diverse demands of disabled people. We opted to develop one broad taxonomy of the findings instead of creating individual taxonomies for each research question. The aspects of the taxonomy illustrate the answers to the first (user groups and assistive services), second (user-centered design of composite services), and third (composition approaches) research questions posited in Section IV. However, we split the taxonomy into two figures for readability and clarity purposes (Figures 21 and 22). In particular, the taxonomy classifies and arranges the types of disabilities and services, user groups, user interaction mechanisms, and service composition methods covered in the selected articles. The taxonomy may be extended in the future to incorporate further knowledge and findings.

One of the key contributions of our survey is the development of an accessible service composition taxonomy for people with disabilities. The taxonomy is designed purposefully to be concise, including a limited number of classes and characteristics, since an overly complex taxonomy would be hard to understand and use and, therefore, often be less counter-productive. We argue that our taxonomy brings about several advantages such as the following:

- The taxonomy organizes our knowledge of service composition users, languages, technologies, integration approaches, and tools tailored to accommodate the needs of disabled people. Through this taxonomy, it is also possible to study the relationships between the various classes and hierarchies of accessible service composition.
- The taxonomy facilitates the understanding and analysis of this somewhat complex domain (i.e., service composition) for fellow researchers and practitioners. For example, the main classes of user-service interactions and deployed technologies in the presence of disabilities can be easily perceived from the taxonomy.
- The taxonomy would enable researchers to identify the existing gaps with respect to the conceptualizations and define novel research directions in this area (i.e., service integration for disabled users).
- Researchers in similar or related fields (e.g., service selection and composition in cloud computing, IoT) are invited to revise and extend the proposed taxonomy to include other relevant conceptualizations and thereby constitute an all-rounded understanding of service composition in several emerging domains (e.g., cloud computing and IoT) for people with special needs.

Figures 21 and 22 depict our proposed taxonomy of accessible service composition, where Figure 21 shows users and services' aspects of the taxonomy, and Figure 22 depicts the composition aspects of the taxonomy. The essential findings from the articles are represented in the hierarchical levels of the taxonomy. The taxonomy entails seven main swimlanes (showed as dotted lines in the taxonomy), where each swimlane represents (1) target user groups and their disabilities, (2) users-services interaction mechanisms, (3) assistive services and technologies, (4) end-user composition, (5) service descriptions, (6) composition approaches, and (7) execution environment of composite services, respectively. Each swimlane incorporates several general categories that are color-coded in gray. Under each category, we incorporate sub-branches to provide further details. Some sub-branches exhibit lower-level details. Wherever appropriate, the levels are arranged in an ascending order depending on the occurrence percentage. In brief, we provide an overview of the main categories of swimlanes.

Users swimlane: This swimlane incorporates three interconnected branches, including the user groups that were targeted by the accessible SOA solutions, the genre of impairments hindering disabled users, and their involvement in the service design process. The latter category (service design) presents the functional and social accessibility features that must be implemented to assist people with disabilities to consume assistive services.

User-service interaction of swimlane: This swimlane details the modes of interaction, input devices, and output devices exploited to enable the interaction between disabled people and assistive composite services.

Assistive services of swimlane: This swimlane sheds light on the types of assistive services composed and the technologies used to access the functionalities of those services. We extend this swimlane to include quality of service factors, including the accessibility and functional properties that were considered when evaluating the quality of composite services. The usual factors for the functional quality of services were assessed such as the response time, cost, and scalability. However, for the accessibility factors, the focus was on the type of disabilities supported and the user interface considerations to facilitate user-service interaction.

End-user composition of swimlane: In this swimlane, we tried to capture the end-user composition aspects, including the users (e.g., professional developers and end-user programmers) and tools (e.g., SM4All) that were employed to facilitate the composition of accessible services for people with special needs. The second branch lists the application areas for which accessible services were created.

Service description of swimlane: This swimlane includes two branches. The first branch provides technical details about the service languages, messaging protocols, and interaction styles used to implement the assistive services. However, the second branch describes the semantic languages and ontologies used to add semantic annotations about the accessibility aspects of services (e.g., impairment type and user profiles).

Composition approaches of swimlane: This swimlane fuses multiple sub-branches detailing components (e.g., data and logic), service selection (e.g., design or runtime), target composite services (e.g., mashup), service binding (e.g., runtime), automation level (e.g., semiautomatic), composition notations (e.g., text or visual), paradigms (e.g., functional and rule-based), patterns (e.g., control flow and data flow), mechanisms (e.g., framework and heuristic), views, and algorithms to achieve service composition. Notably, the composition view refers to the major models applied to achieve service composition, including orchestration, workflow, process mashup, and data mashup. In creating this complex swimlane, we relied on the taxonomies suggested in [6, 22, 49].

Execution aspects of swimlane: This swimlane incorporates two key branches. The runtime features of the composition branch list the possible automation models, deployment types, runtime engines used for executing composite services, and the cross-cutting concerns of utilizing the composite services. The knowledge reuse branch summarizes the artifacts (e.g., types of components, data transformation, examples, mapping rules, and process fragments) and techniques (e.g., search and discovery and recommendation) that were reused during the integration of services.

### 5.6. Quality Assessment of Selected Studies

Our final activity in this SLR pertained to conducting a holistic appraisal of the final articles on 17 critical criteria spanning across distinct quality areas as listed in Table 7. We assessed important research aspects and the areas of service composition that are deemed significant to the accessibility of services. Only the first criterion (i.e., publication quality) was scored depending on the ranking of the journal or conference venue in which the study was published. However, all remaining criteria were scored on a 3-point Likert scale, where 0 = NO, 1 = Yes, and 0.5 = Partially fulfilled. Scores were totaled across all quality criteria and then normalized according to the following formula, where “*S*” denotes the normalized score, “min” denotes the minimum score, and “max” denotes the maximum score.

(1) $$\begin{array}{c} S_{\left(normalised\right)}=\frac{\left({S-S}_{\min}\right)}{\left(S_{\max}-S_{\min}\right)}\text{.} \end{array}$$

Table 7 sums up and sorts the normalized quality scores of all studies. The first five marked “^*∗*^'” are the top-rated service composition studies. Moreover, Table 7 reports additional aspects of each study, such as publication venue, type of publication, and the number of citations. The highly cited articles, e.g., [66, 67, 82], ranked 8^th^, 19^th^, and 1^st^, respectively.

## 6. Key Findings and Open Issues

In this section, we revisit our four research questions and answer them based on the evidence presented in Section V. Before delving into the answers to our research questions, we investigate the main research motivations behind the retrieved studies. Moreover, we synthesize the primary research challenges that should receive the highest consideration from the research community.

### 6.1. Research Motivations

In this subsection, we outline the key motivations that drove the research works of the selected articles. The research motivations could be categorized into the following four general groups:

(i) Develop context-aware systems: Context identification is essential to understand the requirements that empower a system to integrate diverse services to achieve the desired or expected outcome. Various efforts were exerted in literature in this direction, such as ambient assisted living and semantic modeling [95], smart assisted living and semantic modeling [96], and SOA architecture to suggest efficient multimodality paths based on the context [73].
(ii) Solve real-life problems of disabled users: Few studies were motivated to develop technical solutions to overcome the fundamental challenges faced by the disabled or older people. The addressed problems spanned across different domains and the problems are listed as follows:
  - Mobility or tourism: For instance, a microservices platform was proposed where mobility as a service is provided to elderly and disabled citizens to produce personalized routes [70]. Another similar study [77] proposed a framework that can help users with mobility impairments to perform their day-to-day activities and arrange their intercity travel, where the framework gathers the required contents from already existing web services.
  - Shopping: The authors in [108] reiterate the notion of cloud networked robotics where integration of standalone robots and their functionalities is realized to provide seamless support to the daily activities of people with varying disabilities (including the elderly). Six services were considered in the baseline project; however, this study focused on touring services for a physically disabled person in a shopping mall.
  - Healthcare: The authors present a service composition platform and tool to integrate IoT services and heterogeneous IoT products to assist seniors in their daily life activities, i.e., aging in place (AIP) and inside homes [93]. It enables users to create composite services through a graphical tool based on existing atomic services by IoT products. A medication reminder service was produced as a prototype to test the proposed system, which gives reminders to old adults about their medications through various devices, i.e., light, speaker, wrist-band, and smartphone. This study did not explicitly discuss other genres of disabilities except people with mental disorders.
(iii) Devise service composition and selection strategies: Developing service composition or selection strategies is one of the motivating factors for various studies in the literature. Most of these studies can be categorized into static [47, 78, 92, 94], dynamic [67, 68, 71, 84, 107], and hybrid [87, 91, 93, 96, 100], based on selection and composition.
(iv) Build an integration framework, tool, or prototype: Some existing studies aimed to propose frameworks [80, 94] and develop platforms [92], tools [72, 81], and prototypes [73, 76] to facilitate the integration of multifaceted services. For instance, [92] introduces a cloud-based robotic service platform to assist handicapped and elderly people in a robot-friendly environment remotely.

### 6.2. Revisiting Our Research Questions

We now respond to our questions in the next section. Further details are summarized in brief in Table 8, which specifies the uniqueness of the studies and lists their key strengths and weaknesses, respectively.

#### 6.2.1. Research Question 1 (Type of Services and User Interactions)

What are the characteristics of assistive services mashed up to support the regular activities of disabled persons?

Our selected studies are composed mainly of Web and IoT services to realize the promising vision of smart homes/spaces and ambient assisted living. Remarkably, most articles were composed of smart services, sensors, and devices to assist two end-user groups, namely, seniors and people with various disabilities. This makes sense since both user groups share common characteristics (e.g., a decline in their physical and cognitive abilities). It was observed that impaired users accessed services using mainly smart mobile devices. User inputs were provided through voice commands and tactile interactions, while outputs were delivered through screen displays and voice user interfaces. Technically, there was a lack of specification of service description languages and standards. Only about 40% of services were annotated semantically. Moreover, there was an evident lack of using semantic technologies to specify and incorporate accessibility features into services. Generally, accessible services were not added to a common discovery repository for reuse by other researchers and practitioners, which resulted in restricted knowledge sharing and reproducibility. Despite the prevalence of cloud technologies, we were surprised to learn that most services were executed in on-premise environments.

#### 6.2.2. Research Question 2 (Inclusive Design of Services)

To what extent are disabled users involved in designing composite services?

Previous works (e.g., [82]) enumerate the benefits of including participants with disabilities in the design and acceptance of accessible and assistive technologies. However, only 22% of the studies in our survey involved end-users in their SOA studies. Moreover, their validations suffered from two shortcomings, (1) the number of participants was relatively small, and (2) gender-specific characteristics were overlooked in the design process. The adoption of a user-centered design methodology to create inclusive services was lacking from most studies. The results are therefore barely generalizable. While the functional needs (e.g., interaction mechanisms) of service design were discussed, the social aspects (e.g., social appeal) of accessible design were largely overlooked.

#### 6.2.3. Research Question 3 (Service Composition Approaches)

What are the service composition approaches and algorithms developed to incorporate the needs of people with disabilities and accessible design guidelines while creating new assistive services?

The requirements of disabled persons were poorly contemplated and addressed in the selected studies. Although ontologies are well known to enhance interoperability and composition automation, we could not find any accessibility-specific ontology to cater for universal design and accessibility of services. Notably, 38% of the articles did not attempt to model any aspects of disability. Moreover, accessibility modeling focused primarily on the type of disability and interaction modalities. Other important features such as personal preferences, physical context, goals and plans, and service satisfaction were disregarded in the modeling process.

During design and runtime, services were selected to form mashup and business process applications. The composition frameworks and tools relied mainly on text (code and XML) to create the compositions, possibly due to their ability to express complex logic and operations. The composition languages were mainly functional and rule-based in nature. The composition tools were designed for use by software developers and end-user programmers rather than ordinary end-users. SM4All was the most frequent composition tool used to compose universal services. Most of the compositions reused control flow and data flow constructs. Remarkably, no single composition tool supported the development of accessible services via design guidance, performance, and testing.

### 6.3. Research Challenges

Extracting each study's shortcomings gave us more clarity about the potential issues in accessible service composition. In this subsection, we answer our fourth research question.

#### 6.3.1. Research Question 4 (Open Issues)

What are the open challenges hindering the integration of accessible design within service-oriented architecture solutions?

To this end, we classify and summarize the emerging research challenges into the following five main themes: (1) service datasets, (2) semantic description of accessibility characteristics, (3) accessible and universal designs, (4) service composition implementation details, and (5) validation of composition approaches. The below mentioned issues are collated based on our critique of the assembled studies and the authors' perspectives and discussions. Furthermore, we advocate some recommendations to tackle the emerging issues from the studies.

*(1) Assistive Services Datasets*.

- **Availability and accessibility**: Accessible services' datasets are scarce and often kept private except for a few with inadequate information [66, 88, 92, 111]. Arguably, the lack of datasets of accessible services restrains the reproducibility of experiments and limits the reuse of research resources. Therefore, researchers should publish their assistive services on public service repositories for discovery (e.g., through the UDDI standard), reuse, and extension.

*(2) Semantic Description of Accessibility Characteristics*.

- **Comprehensive ontology:** The lack of semantic ontologies dedicated to describing accessible services is one of the prominent challenges. Indeed, some disability aspects were modeled in various domains, such as tourism [72, 77], smart homes [90, 93, 112], ambient and smart assisted living [79, 80, 95, 96], shopping malls [108], e-government [71], and smart cities [107]. However, previous works failed to develop a comprehensive ontology dedicated solely to describing the accessibility aspects of services.

*(3) Accessible and Universal Design*.

- **Social needs and inclusion:** Social challenges, needs, and environment were not considered when designing composite services for people with disabilities. It is essential to go beyond the functional requirements and practical acceptance to operate services and work toward realizing socially designed services that overcome social barriers (e.g., social exclusion and isolation) and achieve social acceptance (e.g., equal opportunities). Few studies showed interest in nonfunctional aspects or social needs such as simplicity with ease of use [83] and social inclusion [77, 85]. Therefore, a social model of disability should be developed and integrated within the current SOA tools.
- **Universal design:** Design for accessible services was overlooked [70, 76, 79, 88, 96]. None of the SOA tools implemented guidelines for universal design. More research should concentrate on annotating services with accessibility features and guidelines to make them ready for consumption by disabled users in smart environments.
- **Engagement of disabled people:** People with disabilities were usually not consulted in developing SOA solutions and accessible services. Our analysis revealed a lack of focus on the requirement analysis for users with special needs [6, 7, 22, 47–50, 52, 53], except in a few instances in which disabled or older adults were involved at some point in the studies [68, 71, 79, 83–85]. Therefore, the requirements identified from disabled people were rather poor and/or incomplete. Our research reiterates the recommendation to involve disabled users in the design of universal and accessible composite services. Equally, there is an urging necessity to engage people with diverse disabilities in the design and validation of service composition approaches. Gender-specific dimensions and needs should also be examined in future studies.
- **Accessibility of design tools:** The development of an accessibility design tool seems nontrivial because no such support tool exists in the literature. Our research suggests the inclusion of design guidance and accessibility checking in SOA tools. This is because service designers and developers lack knowledge and expertise in accessibility design.
- **Accessibility-aware service selection:** Service selection during the composition process was mainly driven by the functional quality of service (QoS) factors, such as efficiency [70, 73, 96], scalability [91, 93, 112], availability [100], and response times [88, 94]. In our view, the inclusion of accessibility QoS properties in the selection process would produce universal composite services that meet the functional and social demands of disabled people.

*(4) Service Composition Implementation Details*.

- **System or prototype development:** The absence of prototypes [73, 100] or implementation details [88, 91, 95] to demonstrate the inner workings of service composition approaches for the disabled users. In various articles, we noticed the lack of technical details concerning the implementation of composition models and algorithms.
- **Heterogeneity:** There is a lack of systems that support heterogeneity in terms of services, people, devices, and QoS parameters in a single platform [85, 87]. Today, we are surrounded by various devices and sensors in an IoT environment, so it is nontrivial to integrate diverse services in such an environment.
- **Autonomous composition:** Although our focus was on the service composition phase, we noticed the lack of using machine learning (ML) models to facilitate the composition activities. A limited number of studies focused on autonomous service composition approaches utilizing recent developments in artificial intelligence. In one study, the authors utilized machine learning for dynamic service provisioning [96]. Similarly, the authors of another study proposed an automatic service integration approach based on AI and ML in the tourism domain [72], where they used a static approach that cannot be extended to big cities. Only a few SOAs support creating automatic service composition [100]. The seamless invocation of services by heterogeneous SOAs remains a significant challenge.

*(5) Validation of Composition Approaches*

- **Disability scenarios:** The definition of user-centric and real-life scenarios was missing in most of the articles [69, 70, 87, 95, 111]. These scenarios and case studies are helpful to facilitate the design and validation of the composition approaches to serve the needs of disabled users.
- **Feasibility and user acceptance:** Evidently, little verification and validation of the composition approaches or produced compositions was conducted [69, 71, 76, 110]. We expect that service composition frameworks and prototypes must undergo both technical feasibility and user acceptance (also establishing the trust) testing to be judged as effective and replicable. The superiority of a new service composition framework should be established through comparative testing against existing frameworks.

**Figure 23:**
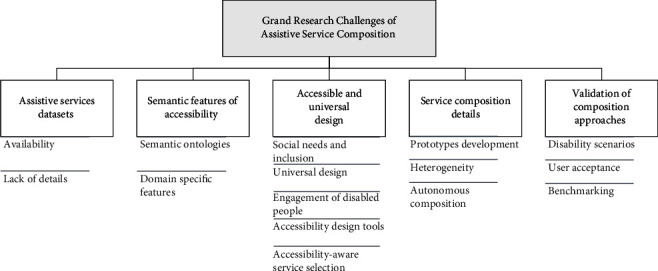
A research agenda for assistive service composition.

Figure 23 summarizes the major research challenges discovered by our systematic survey.

## 7. Study Limitations and Validity Threats

SLRs are quite rewarding when it comes to building a robust understanding of a research problem and answering focused research questions. However, it is imperative to self-criticize the findings and reflect amenably on the limitations of the current SLR. Admittedly, identifying research articles that present service composition solutions tailored towards users with disabilities turned out to be a practical challenge. The research team had to vary the search phrases and resort to manual searches to reach a reasonable number of articles thus ensuring breadth. Although a resource-intense process was conducted to find every possible article, our analysis results were influenced by the quality of the research design and findings reported in the final selection of the articles. We discovered that several details (e.g., technical information about the composition approaches) were missing from the primary studies during the data extraction phases. This could have an influence on the main findings. Another limitation is that our search was limited to six electronic bibliographic databases. On this basis, other academic databases, such as Ei Compendex and Taylor and Francis, were not explored separately. Moreover, our search considered studies published after 2010, which increases the likelihood that we might have missed some important studies because of this decision. Our search was restricted to only peer-reviewed conference and journal articles, which means that interesting findings from short articles and gray literature might have been ignored. Publication and selection bias are major problems that most SLRs suffer from, and we are no exception. This is an outcome of not identifying all available data on assistive service composition.

Threats to the validity of findings affect the quality of systematic literature reviews. Commonly, these threats may arise from four main sources, particularly (1) missing important articles, (2) bias in selecting research articles, (3) erroneous data extraction, and (4) subjective interpretation of evidence. We applied several measures (e.g., PICO and Kitchenham's methodology) to reduce the effects of these threats. The research team explored the major research databases using varied terms through pilot tests before embarking on the final list of keywords (shown in Table 2). Manual search and snowballing techniques were conjugated with automated search so as to not miss any relevant articles. The eligibility criteria for inclusion in our SLR were clearly defined to concentrate on the composition of accessible services for disabled people. The extracted data were revised several times by different authors to ensure their correctness.

## 8. Conclusions and Future Directions

To the best of our knowledge, this is the first research effort to synthesize existing works on service composition for persons with disabilities. This research topic merits investigation and continuance due to several reasons. First, the number of disabled people is estimated at one billion persons, which emphasizes the notion of service design for disability inclusion. Second, other disadvantaged groups that share similar profiles, such as the elderly population, could be greatly assisted by the same SOA-based solutions. Third, the prevalence of IoT devices and smart services opens the horizon for unraveled composition opportunities in smart homes and places.

Our systematic review analyzed and synthesized 38 distinguished service composition studies that were aimed at empowering users with special needs through the provision of assistive services. The primary studies were selected by applying a rigorous search process to articles retrieved from IEEE Xplore, Web of Science, Springer Link, ACM Library, ScienceDirect, and Google Scholar. Our selected publications spanned the range from January 2010 to October 2022 and appeared in major computing and engineering publications, such as Springer and IEEE. Best practices of systematic literature review, such as the PICO model and Kitchenham's methodology, were implemented in this survey.

Our SLR produced the so-called “accessible service composition taxonomy,” which incorporates seven facets, specifically the target users with disabilities, user-service interaction mechanisms, assisted composite services, end-user composition, services' description, composition approaches, and runtime aspects of the compositions. The taxonomy would help composite service developers understand the technical and social requirements of disabled users. Unfortunately, the accessibility of composite services seems to be disregarded, with only a handful of works catering to the needs of people with impairments. Best user-centric and inclusive design practices are not incorporated into the composition of assistive services and technologies. We call upon the research community and practitioners to exert more efforts to (1) engage disabled people in the design of SOA solutions and validation of service compositions, (2) define QoS for accessible services, (3) consider the social aspects of disabilities, (4) provide tool support during the composition phase, (5) and incorporate machine learning advancements to produce more satisfying service compositions that fit the dynamic circumstances of disabled people.

Our future research activities include the design and development of a comprehensive accessibility ontology to facilitate universal service creation and composition. The ontology would detail disability types, personal capabilities, personal characteristics, social considerations, functional requirements, the context of use, interaction mechanisms, and assistive technologies. Such ontology coupled with a service ontology would facilitate the dynamic selection of services during the composition process to meet the demands of the impaired users. Moreover, we plan to propose and implement a machine learning-guided service selection framework to satisfy the dynamic demands of disabled persons while considering the context and accessibility profiles of users.

## Figures and Tables

**Table 1:** Prominent surveys of service composition (spanning 2013–2022) and their major weaknesses (J = journal, C = conference, SLR = systematic literature review, and NS = not specified).

Study	Type	Pub. year	Academic databases and no of articles	Years range	Focus of survey	Major weaknesses
J [22]	Review	2015	NS	NS	Describes a detailed analysis framework of service composition for comparing models, languages, and tools of composition	(i) Survey methodology and article selection unspecified
						(ii) Complex SOA taxonomy of analysis
						(iii) Does not discuss the requirements of disabled people

J [6]	Review	2016	29 articles	NS	Reviews and clusters 29 RESTful service composition approaches using eight features (i.e., composition view, automation, definition, and standards conformance). It also highlights the advantages and drawbacks of each approach	(i) Focuses on RESTful services only; SOAP services were overlooked
						(ii) Does not consider the requirements of people with special needs
						(iii) Composition tools and languages not discussed
						(iv) Survey methodology not reported

J [47]	Review	2013	34 articles	2003–2011	Compares service composition platforms in ambient intelligent environments (AIEs), focusing on their differences and similarities	(i) Survey methodology and article selection unspecified
						(ii) Does not consider disabled persons
						(iii) Selected studies are pretty outdated

J [48]	SLR	2014	34 articles	2009–2013	Explores service composition in cloud environments and assesses major algorithms (e.g., combinatorial, graph, and machine-based) of cloud service composition concerning nine requirements	(i) Does not consider people with special needs
						(ii) Restricted to cloud environments
						(iii) QoS did not consider the accessibility of services
						(iv) Outdated

J [49]	SLR	2017	20 articles from 9 databases	2012–2016	Presents the open issues of service composition in the cloud while considering QoS parameters. Also, it classifies cloud service composition techniques (framework, agent, and heuristic) and suggests improvement areas for cloud service composition	(i) Does not consider people with special needs
						(ii) QoS did not consider the accessible design of services

J [50]	SLR	2018	42 articles from 9 databases	2012–2017	Suggests a taxonomy of service composition approaches in IoT environments while considering functional (e.g., correctness and safety) and nonfunctional aspects (e.g., data-oriented and infrastructure)	(i) QoS factors did not consider the accessibility of services
						(ii) Restricted to IoT/cloud environments

J [51]	Review	2019	NS	NS	Reviews and compares service composition approaches in IoT against numerous criteria, namely, dynamic composition, adaptability, independence and extensibility, distribution, standards, and trust, among others	(i) Comparison criteria are not well explained nor justified
						(ii) Survey methodology and selection criteria were not reported

J [52]	SLR	2018	22 articles from 6 databases	2010–2017	Reviews framework-based, heuristic-based, model-based, and SOA and RESTful-based composition techniques in IoT with a focus on pros and cons. Also inspects the improvements of several QoS parameters such as scalability, execution time, cost, reliability, availability, and response time	(i) Authors focus solely on IoT environments
						(ii) QoS properties do not focus on the needs of disabled users

C [7]	Review	2020	NS	NS	Identifies and assesses 14 prominent service composition languages (e.g., BPEL, BPMN, and AWS step functions) and systems based on several features (e.g., runtime environment, composition model, and IDE)	(i) Survey methodology and article selection unspecified
						(ii) QoS did not consider the user side and accessibility of services

J [53]	Review	2015	25 articles	2002–2015	Surveys and compares ubiquitous application composition approaches, platforms, and metaphors, where the analysis focuses on user involvement and support in the composition process	(i) Survey methodology and article selection protocol unspecified
						(ii) Apart from the PalCom project, there are no references to service composition for disabled users

J [54]	SLR	2021	71 articles	2008–2020	Presents quality of service-aware service composition in the context of cloud computing. Metaheuristics are surveyed in this article where traditional solutions are hybridized through various strategies	(i) Does not discuss the needs of people with disabilities
						(ii) QoS focuses on the technical aspects of service composition rather than usability and accessibility aspects of service usage
						(iii) Results are not presented in a form of taxonomy

J [8]	SLR	2022	100 articles	2007–2020	Presents a classification of service composition and a taxonomy of composition approaches under uncertainty within dynamic environments	(i) No focus on user requirements in uncertain distributed environments
						(ii) Disabled user groups are not considered at all during composition

J [55]	SLR	2022	123 articles from 3 databases	2008–2019	Assesses 17 Web of Things platforms against a set of requirements and characteristics of IoT middleware	(i) Methodology is not thoroughly detailed
						(ii) A shallow analysis of the articles is presented
						(iii) Nonfunctional requirements do not consider accessibility of Web of Things
						(iv) The work is not tailored for disabled users
						(v) Web services were excluded from the analysis

**Table 2:** PICO elements of our SLR.

	Element description	Search keywords/terms
Population (P)	People with disabilities	(Disability OR disabilities OR “disabled people” OR “disabled user” OR “disabled person” OR “disabled group” OR impairment OR impaired OR handicap OR blind OR deaf OR wheelchair OR paralyzed OR crippled OR incapacitated OR “people with limited mobility” OR “special needs”)
Intervention (I)	Service composition (approaches and platforms)	(Service OR workflow OR mashup) AND (Composition OR integration OR combination OR orchestration)
Comparison (C)	Comparison between composition approaches	
Outcomes (O)	Accessible composite services	

**Table 3:** Quality assessment criteria (17 items).

Quality criterion (QC)	Description of criterion
QC1	Quality of publication
QC2	Well-defined research questions
QC3	Clearly explained contributions
QC4	Clearly described motivation scenario of service composition for disabled people
QC5	Clearly presented composition framework/algorithms
QC6	Accessibility design followed (i.e., needs of people with special needs considered)
QC7	Conformance to technical standards, languages, or specifications of service composition
QC8	Clearly stated QoS parameters, including nonfunctional properties of service composition
QC9	Comparison with state-of-the-art service composition methods
QC10	Dataset (of services) clearly defined
QC11	Framework/solution proposed supports the verification and validation of service compositions
QC12	Composition approach accommodates multiple types of disability
QC13	Composition approach tested in different disability contexts
QC14	Composed services/composition tested with end-users
QC15	Threats to the validity of findings discussed
QC16	Research implications (i.e., theoretical and practical) recommended
QC17	Research limitations/challenges of the study clearly highlighted

**Table 4:** Summary of participants in studies (NS = not specified).

Study	Number of participants	Male	Female
[79]	1958	NS	
[76]	31	NS	
[81]	16	NS	
[71]	15	NS	
[83]	12	NS	
[68]	7	5	2
[84]	7	NS	
[85]	1	1	0

**Table 5:** Social aspects examined in selected studies.

Studies	Social needs
[85]	Personalization of services as per the culture and lifestyle of disabled users
[77]	Reduction of social isolation
[83]	Autonomy to survive in isolation; social inclusion
[80]	Individual profiling to help users in social contexts
[92]	Social function services, e.g., inclusion; psychological comfort

**Table 6:** Knowledge reuse (artifacts and techniques) in our selected studies.

Reused artifacts	Reuse technique	Studies
Components	Search and discovery (relational queries)	[71, 73]
	Search and discovery (keyword search)	[72, 74, 77, 80, 85, 87, 90, 93, 97, 100]
	Recommendation (context)	[107]
	Not specified	[66, 68, 83, 84, 92, 108, 111]

Data transformation rules	Search and discovery (keyword search)	[89, 91]

Examples	Search and discovery (graph-structured search)	[99]

Mapping rules	Recommendation (collaborative filtering)	[79]
	Not specified	[78]

Process fragments	Recommendation (context)	[110]
	Search and discovery (relational query search)	[69]

Not specified	Not specified	[67, 70, 75, 76, 81, 88, 94–96]

**Table 7:** Assessment results of selected articles, ordered according to normalized score (^*∗*^top five studies).

Study	Quality criteria (QC)																	Total	Normalized score	Cited	Year	C/J	Publisher
	1	2	4	3	5	6	7	8	9	10	11	12	13	14	15	16	17						
[68]^*∗*^	0.75	1	1	1	1	1	1	1	0	0	1	1	0.5	1	0	1	1	13.25	100.00	74	2013	J	ACM
[83]^*∗*^	0.5	1	1	1	0.5	0.5	1	1	0	0	0.5	0.5	1	1	0	0.5	1	11	80.85	1	2017	J	Inderscience Enterprises Ltd.
[71]^*∗*^	1	1	1	0.5	0.5	1	1	1	0	0	0.5	1	0.5	1	0	0.5	0.5	11	80.85	8	2012	J	ACM
[111]^*∗*^	1	1	1	0.5	0	0.5	0.5	0.5	0.5	0.5	1	0.5	0	0	1	0.5	1	10	72.34	7	2012	C	Springer
[79]^*∗*^	0.75	1	1	1	0.5	0	1	0	0	0	1	0.5	0.5	1	0	0.5	1	9.75	70.21	1	2021	J	Springer
[101]	0.75	0.5	1	0.5	0.5	0.5	0.5	0.5	0	0	0.5	1	0.5	0.5	0.5	1	1	9.75	70.21	0	2022	J	MDPI
[78]	1	1	1	0.5	1	0	1	0.5	0	0	1	0.5	0	0	0	0.5	1	9	63.83	14	2017	J	Springer
[72]	0	0.5	1	0.5	0.5	0.5	1	0	0.5	0	1	0.5	0	1	0	0.5	1	8.5	59.57	11	2015	C	Springer
[66]	0	1	0.5	0	0	1	0	0.5	0	0.5	1	0.5	0.5	1	0.5	0.5	1	8.5	59.57	243	2013	J	Taylor & Francis
[74]	0	1	0.5	1	0.5	1	0.5	0.5	0	0	0.5	1	0.5	0.5	0	0.5	0	8	55.32	6	2016	C	IEEE
[84]	1	1	1	0	1	0	0.5	1	0	0	0.5	0	0	0.5	0	0.5	1	8	55.32	5	2021	J	Springer
[69]	0.75	1	1	0.5	1	0.5	1	0	0	0	0	0.5	0	0	0	0.5	1	7.75	53.19	45	2017	J	MDPI
[110]	0.68	1	1	0	0.5	0	1	0	0	0	0	0.5	0.5	0.5	0	0.5	1	7.18	48.34	25	2015	C	IEEE
[93]	0	0.5	0.5	0.5	1	0.5	0.5	1	0	0	1	0.5	0	0	0	0.5	0.5	7	46.81	7	2018	J	Korea Information Processing Society
[92]	0.75	1	0.5	0.5	0	0	0.5	0.5	0	1	0.5	0.5	0	0	0	0.5	0.5	6.75	44.68	1	2020	J	Hindawi
[89]	1	0.5	0.5	0	0.5	0.5	0.5	1	0	0	0	1	0	0	0	0	1	6.5	42.55	1	2011	C	Springer
[91]	0	0.5	0.5	0.5	0.5	0.5	0	0	0.5	0	1	1	0	0	0	0.5	1	6.5	42.55	8	2012	J	Springer and Science Press of China
[81]	0	0.5	0.5	0.5	0	0.5	0.5	0.5	0	0	1	0	0.5	1	0	0.5	0.5	6.5	42.55	1	2016	J	MDPI
[107]	0	1	1	0	0.5	0	0.5	1	0	0	0.5	0.5	0	0	0	0.5	1	6.5	42.55	9	2016	C	ACM
[67]	1	0.5	0.5	1	0	0.5	0.5	0	0	0	0.5	0	0	0	0	0.5	1	6	38.30	203	2012	J	IEEE
[80]	0.34	0.5	0.5	1	0	1	0.5	0	0	0	0	1	0	0	0	0	1	5.84	36.94	0	2019	C	SciTePress
[115]	0.75	0.5	1	0	0.5	0	0.5	0.5	0	0	0.5	0	0	0.5	0	0.5	0.5	5.75	36.17	4	2022	J	MDPI
[77]	0	0	0.5	0.5	0.5	0.5	0.5	0.5	0	0	0.5	1	0	0	0	1	0	5.5	34.04	8	2010	C	Springer
[97]	0	0.5	0.5	0	0.5	0.5	0.5	0	0	0	1	0.5	0	0	0	0.5	1	5.5	34.04	9	2013	C	Springer
[76]	0.34	1	1	0.5	0.5	0	0.5	0	0	0	0	0.5	0	0.5	0	0.5	0	5.34	32.68	15	2011	C	IEEE
[73]	0.68	0.5	1	0	0	0.5	0	0.5	0	0	1	0	0	0.5	0	0.5	0	5.18	31.32	12	2017	C	IEEE
[99]	0.68	1	0.5	1	0	0	0.5	0	0	0	0	0.5	0	0	0	0	0.5	4.68	27.06	1	2016	C	ACM
[96]	0	0.5	0.5	0.5	0.5	0.5	0.5	0.5	0	0	0.5	0.5	0	0	0	0	0	4.5	25.53	6	2016	C	IEEE
[75]	0	1	1	0	0.5	0	0.5	0	0	0	0	0.5	0	0	0	0.5	0.5	4.5	25.53	11	2011	J	European Alliance for Innovation (EAI)
[87]	0	0.5	0.5	0.5	0.5	0.5	0.5	0	0	0	0	0.5	0	0	0	0	0.5	4	21.28	12	2011	C	Springer
[90]	0	0.5	0.5	0	0	0.5	0	0	0	0	1	0.5	0	0	0	0.5	0.5	4	21.28	12	2012	C	Springer
[85]	0	0.5	0.5	0	0.5	0	0.5	0	0	0	0.5	0	0	0	0	0.5	1	4	21.28	8	2018	J	MDPI
[100]	0.34	0	0.5	0	0.5	0	0.5	0.5	0.5	0	0.5	0	0	0	0	0	0.5	3.84	19.91	11	2010	C	Springer
[108]	0	0.5	0.5	0	0	0	0.5	0	0	0	1	0.5	0	0	0	0	0.5	3.5	17.02	10	2015	J	KICS
[88]	0	0.5	0.5	0	0.5	0	0	0	0.5	0.5	0	0	0	0	0	0.5	0.5	3.5	17.02	6	2014	C	IEEE
[95]	0	0.5	0.5	0.5	0	0	0.5	0	0	0	0.5	0	0	0	0	0	0	2.5	8.51	6	2013	C	Elsevier
[94]	0	0.5	0	0.5	0	0.5	0.5	0.5	0	0	0	0	0	0	0	0	0	2.5	8.51	0	2010	C	Springer
[70]	0	0.5	0	0	0	0	0	0.5	0	0	0	0.5	0	0	0	0	0	1.5	0.00	42	2016	C	IEEE

QC1, publication quality; QC2, research questions; QC3, research contributions; QC4, motivation scenario(s) of disabled persons; QC5, composition framework/algorithm(s); QC6, disability-specific design; QC7, conformance to technical standards of service composition; QC8, QoS parameters/nonfunctional properties; QC9, comparison with composition methods; QC10, dataset (of services); QC11, verification and validation of service compositions; QC12, composition approach supports multiple types of disability; QC13, composition approach tested in different disability contexts; QC14, composition tested with end-users; QC15, threats to validity; QC16, research implications (theoretical and practical); QC17, research limitations/challenges; C/J, conference (C)/journal (J).

**Table 8:** A comparative summary of strengths and weaknesses of the selected studies.

Study	Unique aspects	Strengths	Weaknesses
[95]	A formal system for ambient assisted living application development with a semantic model represented by an upper ontology that uses constructive description logic (CDL)	(+) Ambient assisted living is explored (+) A system to specify a semantic model	(−) No implementation of the proposed composition model (−) No real-life scenarios related to disabled persons

[96]	The proposed model considers context awareness, dynamic service provisioning, and uniform availability of information from heterogeneous devices; machine learning was utilized at multiple phases of service execution	(+) Implementation of model based on WoO platform (+) Semantic ontology model is presented (+) A smart assisted living use case is described	(−) Approach is not well explained (−) The use case is simple (−) No comparison is made with relevant approaches (−) Service composition and selection modules and parameters are not explained

[70]	The components of the proposed mobility platform are implemented as microservices	(+) Concept of mobility as a service is introduced along with crowdsourcing (+) A microservices platform is presented to help elderly and disabled citizens to get personalized multimodal urban routes	(−) No validation of the proposed architecture (−) No real scenarios of disabled persons are presented (−) Service composition and selection are discussed

[73]	The proposed SOA architecture suggests multimodality paths for citizens with reduced mobility and the elderly. The multimodal paths mix bicycle lanes and bike-sharing services in urban environments	(+) A prototype is developed, and a case study is presented (+) User's interface and interaction mechanisms consider the context of use (+) Concepts of multimodality are used	(−) Details related to service composition/SOA are missing (−) Requirements related to users with disabilities are not discussed in detail

[87]	A unique adaptable algorithm adjusts user interfaces as per the device characteristics, such as speech/aural, and visual/touch, based on the user preferences. Service composition is based on an “online synthesis engine” and “offline synthesis engine” to support a dynamic and static set of services for users	(+) Pervasive intelligent home system (i.e., SM4All) for home automation is presented (+) An XML-based format is proposed to define services of home devices (+) Adaptive user interfaces are proposed to support varied settings	(−) No validation of the proposed architecture (−) No real scenario of disabled persons is discussed (−) No prototype is presented

[100]	The presented composition framework exploits service properties to select the most appropriate SOA to enhance the performance. The framework supports automatic, on-the-fly compositions as well as access to a searchable service directory	(+) Support the heterogonous SOA implementations (+) Automatic on-the-fly service composition (+) A case study is presented to validate the results	(−) Study is slightly outdated (−) Prototype is not presented (−) disability-specific requirements are not discussed in detail (−) Different interface options are not considered

[89]	A model-driven engineering (MDE) approach suggests models and related transformations that help form adaptable user interfaces by considering the context of use	(+) Model-driven engineering approach is presented to support adaptable user interfaces (+) Interaction of users using various devices such as desktops, tablets, and smartphones is considered (+) Different modalities are considered (+) Context of use is considered	(−) No validation or proof of concept is presented (−) Disability-specific requirements are not being discussed in detail (−) No disability scenarios are discussed (−) No comparison is made with relevant approaches (−) Transformation rules are defined for adaptable user interfaces

[91]	The presented framework works as an intermediary infrastructure to provide access to edge services according to user needs. The framework enables users to receive composed services based on their preferences	(+) The proposed framework provides access to edge services in a flexible and scalable way (+) Edge services can be composed dynamically (+) User preferences and device characteristics are considered (+) User profiling feature is supported (+) Programmers can easily develop new services using the proposed APIs (+) The framework is validated through experimentation	(−) The proposed framework is not tested with actual disabled users (−) Several issues such as security and performance enhancements are discussed (−) Details related to service composition are missing

[77]	Web services are discoverable based on ontologies, which are presented for domains such as service, transportation, and tourism	(+) Dedicated ontologies are presented to cover various domains such as service, transportation, tourism, personal support, e-learning, and social relations (+) Proposed framework supports the needs of mobility-impaired users (+) Service alignment procedures can be completed with the help of a drag-and-drop GUI (+) Use case scenario is presented	(−) Solution is restricted to a single type of disability (−) Device characteristics are not considered (−) There is no mention of support for context-aware services (−) User interaction with the services, such as input and output from the services, are not defined

[93]	Use of Web of Objects (WoO) concepts for assisted living in smart homes. The presented architecture finds the status of the elderly automatically and enables developers to offer personalized services	(+) A composite virtual object (CVO) representation model is presented for efficient and scalable service composition (+) The CVO-based mechanisms help develop and offer various features and services for smart home environments (+) The proposed CVO-based mechanisms can be dynamic and semiautomatic or predefined and static (+) Implementation and experimental studies are presented (+) A GUI-based composition prototype is developed and presented	(−) Scenarios for various types of disabilities are not presented (−) Comparison with similar works is not carried out in the validation section

[81]	The study introduces a service composition tool that adds voice interaction capabilities to mobile applications to enable disabled users to interact using voice commands	(+) An extension of an existing service composition tool is presented (+) Vocal interfaces are realized in detail (+) Validation of the prototype is conducted	(−) Study is limited to a single tool extension (−) Only voice interaction is considered (−) Study does not present a new service integration framework

[90]	The composition framework is based on the AND_OR search concept. This novel approach makes the search in the partial policy by considering the goal requests. The proposed framework enables users to send new goal requests if the already planned requests have been fulfilled in the smart home	(+) The proposed framework utilizes service composition depending upon the goal-based processes (+) A smart home scenario is described (+) Case study and experimental validations are conducted (+) Multiple interfaces are proposed to cater for the needs of various users	(−) Disability scenarios are not discussed in detail (−) Validity does not cover scenarios of different types of disabilities (−) Processes based on simultaneous goals are not considered

[111]	The use of clustering and classification mechanisms to automatically learn the service capabilities; the proposed algorithm has the capability to perform mining based on the past behavior of services	(+) The proposed approach supports pervasive environments by providing a plug-and-play context awareness (+) Automatic description of services using observational learning (+) An architecture is proposed to integrate the learning capabilities (+) Smart home scenario is explained to validate the proposed approach (+) Simulation of approach is performed (+) Smart home prototype supports users with disabilities	(−) Disability scenarios are not detailed (−) Multiple disabilities are not considered (−) Validity does not cover different types of users (−) The impact of service parameters/QoS on the approach is not discussed (−) The proposed model does not test the complex relationship between the impact of preconditions and effects

[108]	The proposed Web of Objects architecture employs a semantic ontology model to infer knowledge-based intelligence through objects collaboration	(+) Knowledge-driven semantic ontology model is presented (+) A prototype is implemented and presented (+) A mall-based scenario is presented for emergency services (+) Example of a handicapped person is discussed to receive emergency services	(−) No validation scenarios related to disabled users are discussed in detail (−) Multidisability support is overlooked (−) Comparative analysis against existing models is not carried out

[72]	AI techniques, knowledge engineering, and information extraction are utilized to support the integration of tourism services; The uniqueness of the proposed approach comes from the fact that user feedback is considered during the process of automatic composition of web services	(+) Incorporation of ML techniques in integrated tourism (+) A tool is developed to retrieve information from the web automatically (+) OWL-S is used for the semantic description of the services (+) User profiling and feedback are incorporated during the service composition process (+) A use case is implemented for an actual city in Italy (+) Disabled users are supported for various services (+) Multimodality for service selection is considered	(−) Support for multiple disabilities is not mentioned (−) Solution is developed for a specific project, and validation of its generality is not tested (−) The tool is not optimized to handle big data, so it might be challenging to accommodate scenarios of big cities

[66]	The proposed business-technology codesign methodology is unique in the sense that it combines business and technology aspects in an in-home healthcare solution to provide services to the elderly and handicapped users	(+) Field trials are conducted to verify the proposed methodology (+) A prototype is developed (+) End-users are engaged to validate and test the methodology (+) A computational analysis is presented to show the performance of the developed prototype (+) A real-life demonstration is presented (+) Architecture integrates devices, services, and systems	(−) Validation scenarios do not include explicit details about the experience of disabled users (−) Different types of disabilities are not discussed (−) User interface design is not highlighted (−) The proposed methodology is verified for a small in-home setup while complex scenarios are not tested in the study (−) The proposed methodology is also not been tested in business practices

[74]	The approach combines the concepts of mobility navigation lifecycle with the service-oriented architecture and presents both functional and implementation perspectives	(+) Navigation mobility is considered for various types of users, including the disabled (+) An SOA-based architecture is suggested (+) A case study, including indoor and outdoor navigation, is conducted covering real-life scenarios related to disabled persons (+) Multiple types of input/output modalities are included to support disabled persons	(−) The approach is not tested on a large scale (−) The feedback from actual users is not considered (−) No comparison with existing approaches (−) Computational validation of approach is not performed

[85]	The approach finds the best service providers for elderly care through a novel environment, called SCoPE, where several parameters, such as service filtering, composition strategies, and service adherence are utilized. Ranking mechanisms are applied to select services	(+) A novel service selection approach that provides tailored services based on user needs (+) A common language is used to identify services and needs (+) The approach integrates diverse services to meet the social and cultural needs of users (+) A personal profile is created for each user in the context of her lifestyle (+) A case study is presented to validate the proposed idea	(−) The proposed approach is not tested on a large scale (−) Validation and experimental studies are not conducted (−) Disabled scenarios are not included in the study (−) Multiple disability support is not considered (−) Input and output modalities are not discussed

[80]	The proposed framework builds disability ontologies by retrieving knowledge and by solving the conflicts between distributed ontologies; the created ontologies can help people, such as the elderly, disabled, and children, in various services	(+) Specific ontologies are implemented to support service integration for the disabled (+) Medical and social domains are the focus for developing disability ontologies (+) Issues between distributed ontologies are solved using cooperation mechanisms (+) User profiles and files are created to cater for the requirements of individual users (+) The proposed tool helps organizations to create assistive services for disabled persons	(−) Service composition is not explained in detail (−) Disabled user interaction mechanisms and multimodalities are not presented (−) Comparison with existing approaches is not made (−) Validation of the proposed framework is not performed (−) End-users are not involved in the design process

[97]	To solve the problems of dynamicity and diversity in the homecare area, the proposed approach utilizes aspect-oriented approach to design and implement dynamic-workflow-based service composition	(+) Use of aspect-oriented approach to promote dynamicity and diversity (+) The proposed approach promotes enterprise interoperability (+) User preferences are considered in the composition (+) The approach enables caregiver organizations to adjust, add or remove business rules to serve the users of services based on their needs (+) The approach is implemented to show the validity of the idea; (+) The approach can support elderly users and users with impairments	(−) The approach does not present experimental validations related to disabled and impaired users (−) The approach does not provide support for complex business rules (−) JavaScript is used, which makes the approach less flexible (−) New changes are hard to implement (−) User input/output modalities in the context of using services are not discussed

[88]	The algorithm can achieve service composition with autonomy, time efficiency, and good application value in a dynamic environment	(+) Autonomy in robotic service composition (+) Handling of emergency situations (+) Identification of an optimal set of services for robot task planning	(−) The service composition algorithm is abstract with no details (−) The algorithm is analyzed only for emergency events (−) Implementation details are not presented effectively (−) The technique is limited to robotic environments (−) Context information acquisition details are missing

[75]	The platform enables users to interact with the system through various means of communication to achieve the target in-house activities through preordered or ad-hoc activation of a series of multiple services. Multiple services are composed at runtime with given constraints to achieve the desired goals	(+) User-centric and context-aware service composition (+) Target action-driven autonomous services composition (+) Capable of handling multiple users with different abilities and needs (+) Generic algorithm for different types of interfaces and services composition	(−) Absence of detailed service composition algorithm (−) Details of the methodology are omitted (−) No systematic validation of services composition for single and multiple users (−) System acceptability is not evaluated for users with varying abilities

[76]	The system (i.e., smart home for all) is developed for smart homes to help coordinate middleware and user interfaces to perform various in-house activities	(+) A prototype is developed and tested with 31 clients (+) The framework targets both nondisabled and disabled people (+) Multimodal interaction is supported	(−) No systematic validation strategy was adopted (−) Limited experimental analysis (−) Acceptability and usability are not evaluated thoroughly (−) People with disabilities were not tested explicitly

[83]	A generic model-driven approach is introduced to reuse models and services based on the context of physically disabled users. Generality, reusability, and integration of services and user interfaces make the approach unique	(+) Reusability of existing services and UI artifacts based on context (+) Systematic evaluation of services and UI (+) Testing of the framework in a real scenario with people with varying requirements, including disabled (+) Ontology-driven context utilization for service and UI creation	(−) Scenarios are limited to mobile applications (−) Limited type of interactions available for end-users (−) Environmental context is not considered (−) Dependency on the availability of ontology (−) Dynamic characteristics are not determined so statistic ontology is utilized

[69]	The use of ontology makes the graphical-based composition framework generic and unique to integrate and reuse heterogeneous IoT products for target activities of people with mental disorders	(+) Integration and reuse of heterogeneous IoT products for different scenarios (+) Platform is built on top of SOA that support modularization, composition, and model-driven implementation (+) New IoT products or devices can be easily integrated with a graphical-based system (+) Web-based interfaces for service composition tool on top of the proposed platform	(−) Lack of systematic validation of the composition model and prototype (−) Unrealistic assumptions were considered, e.g., availability of IoT sensors and equipments in homes (−) Absence of comparative results analysis (−) IoT products are not integrated dynamically (−) One service (medication reminder) is supported by the prototype (−) Details of context identification in the environment are omitted

[68]	Dynamicity in services composition based on the context to achieve the desired target; the service composition approach implements context awareness and manages contingencies, leverages heterogeneous devices, and empowers users	(+) Dynamic composition of services in smart homes based on the context (+) Service/devices are added or removed on an ad-hoc basis (+) Various quality factors are met, e.g., usability and efficiency (+) The system is tested and validated with end-users (+) A detailed methodology and scenarios are presented	(−) The proposed system is not accessible outside home premises (−) Environmental threats are not discussed (−) Blindness and other disabilities scenarios are not studied (−) Comparative analysis is not carried out with other systems

[110]	A service-oriented workflow-based mobile cloud middleware framework is presented to reduce computation overhead based on a fuzzy set and weight of context schemes. The framework enables real-time service composition from heterogeneous proximal pervasive resources	(+) Intelligent services selection and migration from mobile to cloud platform on an ad-hoc basis	(−) QoS is not considered (−) No validation of the composition model (−) Comparison to similar approaches is not presented (−) The proposed system is not tested with real users (with any kind of disabilities)

[67]	Initial work in describing robotic service composition in the context of cloud infrastructure to provide seamless support in daily activities for people with varying disabilities (elderly and disabled)	(+) First is to introduce the notion of cloud networked robotics (+) The concept of generalization of standalone robotic functionalities is used to generate diverse solutions (+) Challenging issues were highlighted in robotics	(−) Discussion is limited to an existing ongoing project (−) No concrete solution is provided rather an example scenario is discussed

[99]	Semantic integration of publicly available accessibility-related web services where a scenario of a visually impaired person to use a screen reader with already defined preferences is described	(+) Alternative approach for providing assistive technologies to the end-user (+) Automation of semantic service composition in the domain of accessibility	(−) Absence of detailed description of the proposed tool (−) Composition strategy is abstractly defined (−) No concrete algorithm for composition is presented

[94]	An e-service architecture is suggested to integrate ambient assisted living with ICT solutions for the elderly with physical and mental disabilities, such as Alzheimer and mild dementia	(+) Design consideration for seniors (+) Exploits existing ICT solutions for the elderly	(−) No concrete solution is presented (−) An abstract architecture is suggested but with no specifics/technical specification

[71]	The composition of context-aware user interfaces as services with existing web services. Context of user interface as services becomes context for other services using a specialized modeling language devised to meet needs of color blind and visually impaired users	(+) Easy integration of user interfaces as services with existing web service management system (+) Context is inferred from user interface services (+) Generic description of user interfaces as services through user interface description language (+) Prototype is tested/evaluated with end-users (+) Functional and nonfunctional evaluation (+) No limit on the number or type of services that can be integrated/composed	(−) Validation is limited to a few people (−) Prototype is tested with a limited number of interfaces

[107]	The service composition model enables autonomous interaction and composition of services in pervasive environments during runtime. The services are defined using a dedicated semantic ontology	(+) Services are pushed to users (no need to request) (+) New service creation is based on the interaction between services (+) Feasibility analysis through proposed prototype with three applications (+) Generation of many composite services to be pushed into the environment (+) Learning interaction patterns based on previous interactions	(−) Inability for the interaction of services with different application domains (−) User-defined semantics while defining the service components at a fine-grained level (−) Service components need to be defined for a diverse set of IoT devices in the environment (−) Composition may result in producing unmeaningful services for end-users

[84]	A generalized service composition approach to produce customized assistive tasks for different target users based on their profiles where abstract workflows are mapped to concrete plans through normative reasoning	(+) Semiautomatic approach (through abstract workflows descriptions) (+) Validation scenario with real end-users (+) Reliability and adaptability to uncertain situations (+) Concrete algorithms are defined	(−) Small number of end-users (7) are considered for validation (−) Limited type of modalities for interaction (i.e., vocal and text) (−) Deployment seems difficult due to the unavailability of ambient assisted living environment

[92]	A cloud-based robotic service platform assists handicapped and elderly people using path planning service and resource matching services in a robot-friendly environment. The proposed approach includes a service-matching strategy to find and delegate tasks to suitable robots based on user requests	(+) Proposed a universal three-layer robot service platform based on SOA (+) Realization of robotic cloud infrastructure for assisting elderly and handicapped people (+) Resource and computation efficient strategy (+) Semiautonomous robot service provision to disabled people	(−) System is not verified with end-users (−) Algorithmic details of robot path planning and resource matching strategy are missing (−) Diversity of robotic services to be integrated is overlooked (−) Environmental constraints are not defined

[79]	The approach provides ambient assisted living services to elderly people with cognitive impairment where services are identified and selected based on predefined semantics	(+) Recommendation of services and tools based on semantic and statistical similarity computations (+) Integration of matchmaking module with an existing AAL platform, i.e., IN-LIFE (+) Involvement of a large number of end-users in the evaluation (+) Concrete rule-based strategy is defined (+) Conformance to existing standards for semantic knowledge representation	(−) Diversity of end-users with varying disabilities (−) Predetermined/restricted semantic rules for service selection and composition

[78]	The system module automatically configures user interfaces of the target devices based on end-user requirements. The matchmaking mechanism is generic and scalable, in contrast to the existing solutions based on propositional statements, which is based on ontological representation of preferences of people with special needs, rather than rigid descriptions	(+) Validation of the proposed matchmaking strategy for automatic UI configuration by a human expert (+) Scalable approach to meet diverse user demands and heterogeneous accessibility aids (+) Transparency for the developers and end-users (+) Concrete rules are defined with example scenarios	(−) Configuration is yet to be explored (−) Configuration solution with matching inference mechanism needs to be improved (−) Proposed system is not tested directly with end-users, but rather evaluated through an emulator (−) Dependency of specifying user profile/preferences

[101]	The proposed model incorporates AI and IoT that can help in the development of IoT applications in a broker-oriented architecture where intelligent agents exchange information through shared memory	(+) Extends the BPEL markup language (+) Application scenarios for the disabled have been discussed including healthcare, home automation, and system integration (+) Aspects such as dynamism, limited expressiveness, and the lack of continuity in the development process have been discussed in detail	(−) The model cannot deal with fast-changing interaction structures (−) The proposed model has been designed solely for the IoT based systems where the expected message traffic will be low than moderate latency requirements (−) The proposed model has not been compared with other existing models in terms of performance and efficiency

[115]	The presented architecture is based on planes and layers that help to cater for the aspects related to the technologies used and group the different tasks. Additionally, their proposed approach tries to solve the problem of heterogeneity and dissemination of information and services related to tourism services scenarios	(+) A unique architecture based on a novel idea of planes and layers (+) The solution solves the problem of heterogeneity and dissemination of information and services (+) They make use of already-established platforms (+) Real scenarios have been used to test the proposed architecture (+) A prototype has been implemented (+) The idea of social sensing has been explored	(−) The model has not been tested for disability scenarios (−) User input/output modalities in context of using services is not discussed (−) Disabled users aspects have not been detailed or discussed

## Data Availability

The data supporting this systematic literature review were extracted from previously reported studies, which have been cited. The processed data are available from the corresponding author upon request.
